# Network meta-analysis of the effects of combined exercise and vitamin intervention on insulin resistance and related indicators in patients with type 2 diabetes

**DOI:** 10.3389/fnut.2025.1608634

**Published:** 2025-11-19

**Authors:** Fangquan Deng, Yin Ji, Haijun Kong, Yebiao Fu, Hanqiao Zhang, Junting Zhang

**Affiliations:** 1 College of Physical Education, Kashgar University, Kashgar, China; 2 Department of Physical Education, Shandong Jiaotong University, Jinan, China; 3 Department of Aviation Security, China Civil Aviation Flight Academy, Guanghan, China; 4 School of Aviation Sports, China Civil Aviation Flight Academy, Guiyang, China

**Keywords:** type 2 diabetes, insulin resistance, vitamin intervention, exercise intervention, network meta-analysis

## Abstract

**Objective:**

This study aimed to evaluate the effects of vitamin supplementation, exercise, and their combined interventions on insulin resistance and related outcomes in patients with type 2 diabetes (T2D). Additionally, it examined the dose–response relationships between vitamin dosage, exercise intensity, and improvements in insulin resistance.

**Methods:**

Relevant studies investigating the impact of vitamin supplementation and exercise interventions on insulin resistance in T2D patients were systematically retrieved from authoritative domestic and international databases, followed by comprehensive synthesis and analysis.

**Results:**

Traditional meta-analyses revealed that both short-term (<12 weeks) and long-term (>12 weeks) interventions significantly improved insulin resistance and related outcomes. The exceptions included vitamin supplementation alone, which did not significantly improve glycated hemoglobin (HbA1c); neither exercise alone or vitamin supplementation alone, which failed to significantly reduce fasting blood glucose; and combined exercise interventions, which had no significant effects on insulin levels. All other interventions yielded significant benefits. Network meta-analysis revealed that, compared with the control group, probiotics provided the greatest improvement in insulin resistance. Vitamin D was most effective at improving HbA1c, whereas vitamin C had the strongest effects on fasting blood glucose and insulin indices. Dose-subgroup analysis indicated that vitamin supplementation up to 2000 IU/day most effectively reduced fasting blood glucose (*p* < 0.01) but had no significant effects on HbA1c or insulin (all *p* > 0.05). A dosage of 2,100–4,000 IU/day produced the most pronounced improvements in HbA1c (*p* < 0.01) and insulin (*p* < 0.05) but did not significantly affect insulin resistance or fasting blood glucose (all *p* > 0.05). Supplementation at 4100–7500 IU/day yielded the greatest improvements in insulin resistance (*p* < 0.01) but had no significant effect on HbA1c (*p* > 0.05). Exercise interventions with an intensity of ≤4 METs, performed three times per week, significantly improved insulin resistance, HbA1c, and insulin indices. Sessions lasting ≤60 min produced optimal benefits for insulin resistance and insulin measures, whereas sessions ≤45 min were most effective for HbA1c and fasting blood glucose.

**Conclusion:**

Vitamin supplementation at 4100–7500 IU/day combined with moderate-intensity exercise (approximately 4 METs) performed three times per week with each session lasting 45–60 min, yielded the most favorable improvements in insulin resistance and related metabolic outcomes in T2D patients.

**Systematic review registration:**

https://www.crd.york.ac.uk/PROSPERO/view/CRD420250655264.

## Introduction

According to the World Health Organization (WHO), approximately 180 million people worldwide have diabetes, and this number is projected to rise to 360 million by 2030. Each year, diabetes contributes to an estimated 2.9 million deaths, making it a major global public health challenge (1). Type 2 diabetes mellitus (T2DM), also known as non–insulin-dependent diabetes mellitus (NIDDM), accounts for more than 90% of all diabetes cases and occurs predominantly in middle-aged and older adults (1).

Insulin resistance (IR), a key pathophysiological feature of T2DM, is closely linked to the development and progression of the disease. Most patients with T2DM present with IR, which is defined as a diminished responsiveness of insulin-target tissues to elevated insulin levels. This impaired response reduces the body’s ability to regulate blood glucose, leading to chronic hyperglycemia. Over time, persistent hyperglycemia induces *β*-cell dysfunction and failure, resulting in chronic hyperinsulinemia and ultimately progressing to T2DM (2, 3). Importantly, prediabetes represents a transitional stage between normal glucose tolerance and T2DM and serves as a critical warning period (4). Individuals in this stage already exhibit insulin resistance and *β*-cell dysfunction, both of which contribute to the onset and persistence of T2DM. Insulin resistance and impaired insulin secretion jointly characterize this stage. Furthermore, IR and T2DM are strongly associated with obesity (5).

Current interventions for prediabetes and T2DM include vitamin supplementation and exercise. Vitamin supplementation plays an important role in suppressing inflammation, controlling blood glucose, and enhancing insulin secretion. Exercise, considered one of the five cornerstones of T2DM management, is valued for its affordability, convenience, and systemic health benefits. Research has shown that exercise improves obesity-related outcomes and reduces glycated hemoglobin (HbA1c), total cholesterol (TC), triglyceride (TG), low-density lipoprotein cholesterol (LDL-C), high-density lipoprotein cholesterol (HDL-C), and fasting insulin (FINS) levels. However, the effects of vitamins on diabetes remain a subject of debate, and the optimal parameters of exercise—such as duration, intensity, and type—have not been clearly defined (1). In addition, although some studies suggest that combining exercise with dietary and lifestyle interventions achieves greater benefits in weight reduction and prevention or management of T2DM than exercise alone, the overall effectiveness of these combined approaches requires further validation.

To address these gaps, the present study employed a network meta-analysis to evaluate the effects of vitamin supplementation, exercise interventions, and their combination on insulin resistance in patients with T2DM. Furthermore, a dose–response meta-analysis was conducted to identify the optimal dosage of vitamin supplementation and exercise parameters, aiming to provide evidence-based recommendations for the management of insulin resistance in T2DM patients.

## Research subjects and methods

This study followed the PRISMA 2020 guidelines for systematic reviews and meta-analyses and has been registered on the international PROSPERO platform, with the registration number CRD420250655264.

### Literature search strategy

A comprehensive search was conducted in both domestic and international academic databases, including PubMed, Web of Science, Embase, Scopus, Cochrane Library, the China Biomedical Literature Database, CNKI, the Wanfang Database, and the VIP Database. The search was limited to publications in Chinese and English, with a cutoff date of December 24, 2024. The Chinese search terms included: vitamin and exercise or training or “physical activity and type 2 diabetes and insulin resistance.” The English search terms included: “insulin resistance” OR “Insulin resistance” OR “Metabolic syndrome” OR “insulin resistance factor uremia” OR “Pseudoacromegaly with Severe Insulin Resistance” OR “HAIR-AN syndrome” AND “type 2 diabetes” OR “Diabetes Mellitus Type 2” OR “Maturity-Onset Diabetes of the Young” OR “Type 2” OR “T2DM” AND “vitamin” AND “sport” OR “exercise” OR “physical activity” OR “training.” Please refer to Appendix 2 for the specific process.

### Inclusion and exclusion criteria

Studies were included according to the PICOST framework. Eligibility criteria: Population (P): Patients diagnosed with T2DM, regardless of age, sex, or disease duration. Patients with or without additional complications were considered. Intervention (I): Vitamin supplementation (e.g., vitamin D, vitamin E, folic acid), with no restrictions on dosage or frequency. Exercise interventions (including aerobic exercise, resistance training, traditional exercise, and aquatic exercise), with no restrictions on frequency, duration, or type. Combined interventions of exercise plus vitamin supplementation, with no restrictions on vitamin type, exercise modality, frequency, or dosage. Comparison (C): Control groups received placebo supplementation without vitamins and maintained usual physical activity. Outcomes (O): Insulin resistance and related metabolic indicators. Study design (S): Randomized controlled trials (RCTs), with no language restrictions. Time frame (T): No restrictions on intervention duration.

Exclusion criteria: Studies were excluded if they met any of the following conditions: reviews, theoretical studies, animal experiments, or conference abstracts; interventions not involving exercise, vitamin supplementation, or their combination; or data reported without means ± standard deviations.

### Literature screening and data extraction

Two independent researchers screened the literature according to the predefined inclusion and exclusion criteria. A third researcher resolved any disagreements. The selection process included the following steps: 1. Identification and removal of duplicate records. 2. Title screening to exclude survey studies, reviews, studies with unrelated interventions, and animal experiments. 3. Abstract and full-text screening to exclude studies with incomplete data, unclear interventions, or undefined outcome measures; and 4. Verification of eligible studies was performed by a third researcher. Final confirmation of the included studies and extraction of relevant data. Data extraction included the following: study characteristics: first author and year of publication. Participant information: Age, sex, and sample size of the experimental and control groups. Study design: type of intervention, duration, frequency, and evaluation indicators. Outcomes of interest: IR, fasting blood sugar (FBS), HbA1c, FINS, TC, TG, LDL-C, and hHDL-C. To ensure consistency, all outcome measures were converted to international standard units. For example, blood glucose values reported in mg/dL were converted to mmol/L. Insulin resistance was assessed via the homeostasis model assessment of IR (HOMA-IR).

### Quality assessment

Two researchers independently evaluated the methodological quality of the included studies via the Cochrane risk of bias assessment tool. The assessment covered seven domains, including random sequence generation, allocation concealment, and blinding. A third researcher resolved any disagreements.

### Statistical analysis

This study used Stata software to conduct a meta-analysis of intervention effects on the outcome measures from the included studies. All primary indicators—including IR, FINS, FBS, and HbA1c—were treated as continuous variables. The network meta-analysis employed Stata to perform traditional meta-analysis and dose–subgroup analyses. R software (version 4.4.1) was used to test model consistency, assess convergence, generate cumulative ranking results, and evaluate publication bias.

### Assessment of evidence quality

This study applied the GRADE approach to evaluate the quality of evidence for the included outcomes. The assessment considered five domains: risk of bias, indirectness, inconsistency, imprecision, and publication bias. On the basis of these domains, each outcome was graded as very low, low, moderate, or high quality.

## Results

### Literature selection results

**Figure 1:**
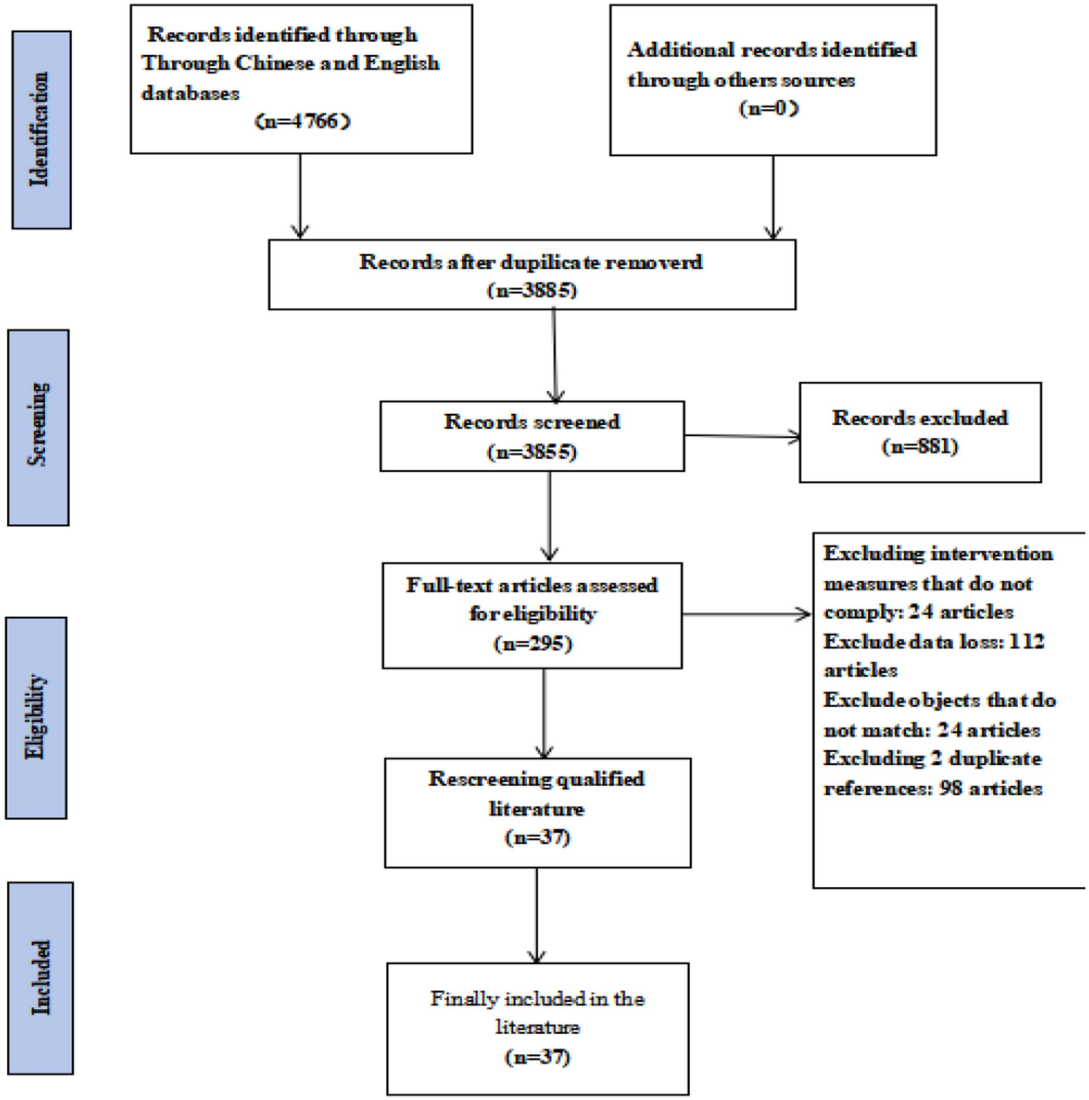
Flowchart of literature screening.

A total of 4,766 articles were initially retrieved from domestic and international databases. After removing duplicates, animal studies, and other ineligible records, 295 articles remained for preliminary screening. Following full-text review, 37 studies met the inclusion criteria (Figure 1). These included 5 two-arm trials, 6 studies with dual interventions, and 12 studies with multiple interventions.

The baseline characteristics of the included studies are summarized in Table 1. Overall, 2,828 patients with type 2 diabetes were enrolled, with 1,672 participants in the test group (T) and 1,156 in the control group (C). Interventions included vitamin C, trace element, vitamin D, aerobic exercise, resistance training, unsaturated fatty acid, combined exercise, combined vitamin supplementation, and exercise plus vitamin supplementation. All interventions lasted at least 8 weeks.

**Table 1:** Basic characteristics of included studies.

First author and year	Number of cases		Intervention measures		Intervention cycle	Outcome indicators
	EX	CON	EX	CON	(week)	
Meng Qing 2018 (25)	40	40	B, N	A	12	1, 2, 3
Fatemeh 2020 (26)	23	23	D, K, R	A	16	1, 2, 4, 5, 6, 7
Amini Lari, Z 2017 (22)	15	15	B, C, N	A	12	1, 4
El-Aal 2018 (27)	10	10	F, E, O	A	12	1, 2, 3, 4, 5, 6, 7
Mogha, M 2019 (21)	10	10	B, C	A	10	1, 4
Aguayo 2020 (28)	12	12	D, O	A	12	1, 4, 7
Sun, X 2023 (12)	15	15	D, B, P	A	12	1, 2, 3, 4
Raygan 2018 (29)	30	30	S	A	12	1, 2, 3
Darmian 2021 (30)	11	10	I, B, T	A	8	1, 2, 3, 4, 7
de Oliveira 2011 (31)	25	26	U, E, O	A	16	1, 4, 5, 6, 7
Hua, Limei 2020 (32)	54	54	G, Q	A	12	1, 2, 3, 4, 5, 6, 7
Amaravadi 2024 (19)	75	71	B	A	12	1, 2, 3, 4
Yavari, A 2012 (33)	20	20	B, C, N	A	52	2, 3, 7
Ali, Amani 2023 (34)	45	45	H	A	24	1, 2, 3, 4, 5, 6
Michelin,2018 (20)	8	8	M	A	12	1, 2, 3, 4
Shabkhiz 2021 (35)	10	10	B	A	12	1, 3, 4
Rehman 2017 (36)	51	51	B	A	25	1, 3, 4
Farrokhian 2016 (37)	30	30	L	A	8	1, 3, 4, 5, 6, 7
Hakami, M 2024 (38)	34	34	B	A	12	2, 3
Terauchi, Y 2022 (39)	97	107	N	A	12	1, 3, 4, 5, 6
Jeon, Y K 2020 (40)	21	14	N	A	12	1, 2, 5, 6
Hodaei, H 2019 (41)	33	23	I	A	10	1, 2, 3, 4
Khalili, L 2019 (42)	20	20	J	A	8	1, 2, 3, 4
Croche 2012 (43)	14	14	G	A	4	1, 4, 5, 6
Li Faxin 2020 (44)	40	40	N	A	12	1, 2, 3, 5, 6, 7
Yang Shigui 2020 (15)	40	30	D, B, P	A	28	1, 2, 3, 4
Rostamian 2024 (14)	10	10	D, B, P	A	8	1, 3, 4, 5, 6, 7
Salarinia 2023 (45)	10	10	D, B, P	A	8	1, 3, 4, 5, 6, 7
Dadrass, A 2019 (46)	12	12	D, C, P	A	12	1, 2, 3, 4, 5, 6, 7
Hoseini, R 2022 (47)	10	10	D, B, P	A	8	1, 2, 3, 4
El Hajj 2020 (48)	45	43	D	A	24	1, 2, 3
Zhou W, P 2020 (49)	45	44	B	A	12	1, 3, 4
El-khodary, 2022 (50)	50	50	H	A	12	1, 2, 3, 4
Rezagholiz 2018 (51)	20	20	D	A	8	1, 2, 3, 4
Baziar, N 2014 (13)	43	44	D	A	8	1, 3, 4
Safarpour, P 2020 (52)	42	43	D	A	8	1, 2
Lai, M H 2008 (53)	10	10	G, O	A	24	1, 2, 3, 4

Intervention measures: A: control group, B: aerobic exercise, C: resistance exercise, D: vitamin D, E: vitamin E, F: vitamin C, G: unsaturated fatty acids, H: vitamin K, I: curcumin, J: probiotics, K: dietary supplements, L: trace elements, M: interval exercise, N: combined exercise, O: combined vitamin, P: vitamin combined exercise, Q: fatty acid combined exercise, R: vitamin combined dietary supplements, S: vitamin combined probiotics, T: exercise combined curcumin, U: vitamin B. Outcome measures: 1. Insulin resistance model (HOMA-IR), 2. Glycated hemoglobin (HbA1c), 3. Blood glucose (FBG), 4. Insulin, 5. Low density lipoprotein cholesterol (LDL-C), 6. High density lipoprotein cholesterol (HDL-C), 7. Triglycerides (TG). EX, experimental group; CON, control group.

### Risk of bias assessment

**Figure 2:**
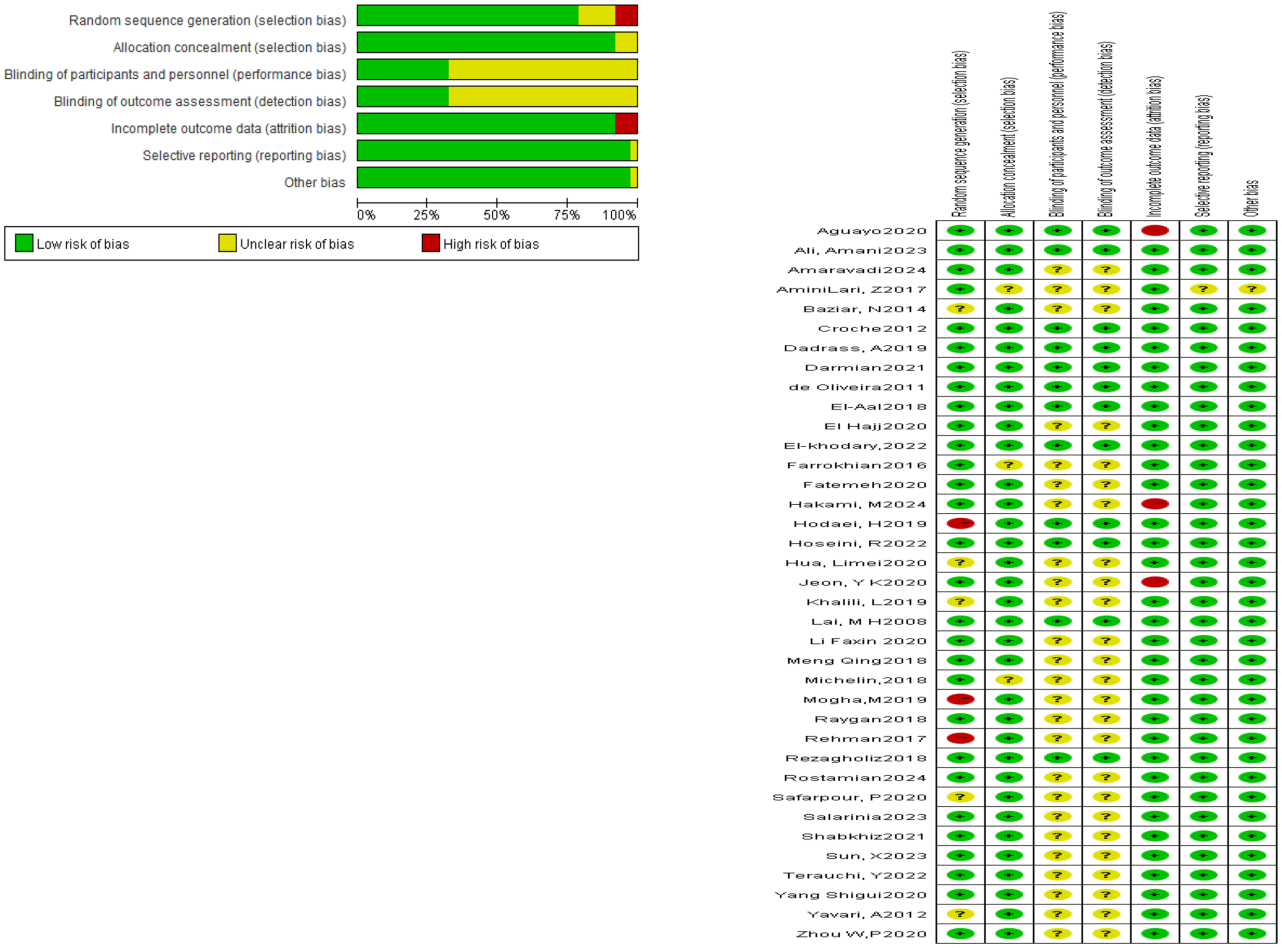
Risk assessment table for inclusion of literature bias.

All 37 included studies reported the use of randomization. However, 31 studies did not provide detailed descriptions of the randomization method. One study used computer-generated randomization, one applied block randomization, one adopted sealed envelopes, and one used a random number generator. Among the included studies, three explicitly implemented single-blinding, nine applied double-blinding, and the remaining studies did not mention blinding procedures. Three studies presented a potential risk of incomplete outcome data. With respect to participant characteristics, 29 studies included patients with comorbidities. The reported comorbidities included coronary heart disease in two studies, hyperlipidemia in one study, and hypertension in one study. The detailed results are presented in Figure 2.

### Traditional meta-analysis results

A total of 33, 21, 26, and 28 studies were included to assess the effects of interventions on IR, HbA1c, FBS, and FINS, respectively. Overall effect analyses indicated significant heterogeneity across studies (IR: *p* < 0.01, I^2^ = 90.8%; HbA1c: *p* < 0.01, I^2^ = 85.1%; FBS: *p* < 0.01, I^2^ = 93.8%; FINS: *p* < 0.01, I^2^ = 86.4%), suggesting the use of a random effects model. The results demonstrated that, compared with controls, interventions significantly reduced IR and associated metabolic indicators: IR: SMD = −1.217, 95% CI [−1.490, −0.945], *p* < 0.01; HbA1c: SMD = −1.061, 95% CI [−1.315, −0.807], *p* < 0.01; FBS: SMD = −1.122, 95% CI [−1.505, −0.740], *p* < 0.01; FINS: SMD = −0.908, 95% CI [−1.152, −0.665], *p* < 0.01.

Subgroup analyses revealed that single exercise, combined exercise, single supplementation with vitamins, unsaturated fatty acids, or trace elements, and combined interventions with vitamins, unsaturated fatty acids, trace elements, or exercise significantly reduced IR (SMD range: −0.854 to −1.916, all *p* < 0.05). Single exercise significantly reduced IR (SMD range: −0.854 to −1.916, all *p* < 0.05). Single exercise, combined exercise, single supplementation, and combined interventions significantly improved HbA1c (SMD range: −0.957 to −1.534, all *p* < 0.05). However, combined supplementation with vitamins, unsaturated fatty acids, and trace elements alone did not significantly affect HbA1c (SMD = −0.247, 95% CI [−1.169, 0.676], *p* > 0.05). Single exercise and single supplementation did not significantly reduce FBS (SMD = −0.684, 95% CI [−1.543, 0.174], *p* > 0.05; SMD = −1.063, 95% CI [−1.505, 0.621], *p* > 0.05). In contrast, combined vitamin/unsaturated fatty acids/trace elements, combined exercise, and unsaturated fatty acids/trace elements plus exercise significantly reduced the FBS (SMD range: −0.598 to −5.001, all *p* < 0.01). Single exercise, single supplementation, combined vitamin supplementation, and other combined interventions significantly reduced insulin levels (SMD range: −0.715 to −1.718, all *p* < 0.05), whereas combined exercise alone did not significantly affect insulin (SMD = 0.024, 95% CI [−0.521, 0.568], *p* > 0.05).

Intervention duration analysis revealed that short-term (<12 weeks), medium-term (12 weeks), and long-term (>12 weeks) interventions significantly reduced IR, insulin, and HbA1c (all *p* < 0.01). Interventions shorter than 12 weeks or longer than 12 weeks also significantly reduced FBS, whereas 12-week interventions did not significantly affect FBS (SMD = −0.479, 95% CI [−1.094, 0.135], *p* > 0.05). The detailed results are presented in Table 2.

**Table 2:** Subgroup analysis of exercise combined with vitamin intervention on insulin resistance and corresponding indicators.

Index	Compare types	Effect value	*P*
Insulin resistance	Individual exercise	−0.854 (−1.312, −0.396)	0.000
	United Movement	1.347 (−2.424, −0.269)	0.014
	Individual vitamins	−1.271 (−1.677, −0.865)	0.000
	Combined vitamins	−1.909 (−3.621, −0.196)	0.029
	Vitamin combined exercise	−1.916 (−3.010, −0.823)	0.001
	<12 weeks	−0.949 (−1.289, −0.610)	0.000
	12 weeks	−0.990 (−1.436, −0.544)	0.000
	>12 weeks	−1.836 (−2.421, −1.252)	0.000
	total	−1.217 (−1.490, −0.945)	0.000
Glycosylated hemoglobin	Individual exercise	−1.163 (−1.598, −0.728)	0.000
	United movement	−0.957 (−1.667, −0.247)	0.008
	Individual vitamins	−1.019 (−1.419, −0.619)	0.000
	Combined vitamins	−0.247 (−1.169, 0.676)	0.601
	Vitamin combined exercise	−1.534 (−2.360, −0.708)	0.000
	<12 weeks	−1.738 (−2.478, −0.999)	0.000
	12 weeks	−0.927 (−1.269, −0.585)	0.000
	>12 weeks	−0.907 (−1.347, −0.467)	0.000
	total	−1.061 (−1.315, −0.807)	0.000
Fasting blood sugar	Individual exercise	−0.684 (−1.543, 0.174)	0.118
	United movement	−0.598 (−1.367, −0.172)	0.000
	Individual vitamins	−1.063 (−1.505, 0.621)	0.128
	Combined vitamins	−5.001 (−6.855, −3.147)	0.000
	Vitamin combined exercise	−2.242 (−3.722, −0.762)	0.003
	<12 weeks	−2.451 (−3.335, −1.567)	0.000
	12 weeks	−0.479 (−1.094, 0.135)	0.126
	>12 weeks	−1.178 (−1.762, −0.593)	0.000
	Total	−1.122 (−1.505, −0.740)	0.000
Insulin	Individual exercise	−0.715 (−1.135, −0.296)	0.001
	United Movement	0.024 (−0.521, 0.568)	0.932
	Individual vitamins	0.907 (−1.239, 0.574)	0.000
	Combined vitamins	−1.718 (−3.382, −0.055)	0.043
	Vitamin combined exercise	−1.704 (−2.715, −0.693)	0.001
	<12 weeks	−1.823 (−2.531, −1.115)	0.000
	12 weeks	−0.551 (−0.857, −0.245)	0.000
	>12 weeks	−0.942 (−1.307, −0.577)	0.000
	total	−0.908 (−1.152, −0.665)	0.000

### Dosage subgroup analysis

This study collected and standardized intervention doses to quantify the effects of vitamin supplementation and exercise on insulin resistance. For fat-soluble vitamin interventions, 0 IU/day served as the reference, and for exercise interventions, 0 MET·hour served as the reference. The study then conducted a quantitative assessment of the relationships between vitamin dose, exercise intensity, and IR in patients with type 2 diabetes. The dose–response meta-analysis revealed that fat-soluble vitamin supplementation up to 2,000 IU/day produced the greatest improvement in fasting blood glucose (FBS) (*p* < 0.01) but had no significant effect on hemoglobin A1c (HbA1c) or insulin levels (both *p* > 0.05). Doses of 2,100–4,000 IU/day optimized improvements in HbA1c (*p* < 0.01) and insulin (*p* < 0.05) but did not significantly affect insulin resistance or FBS (both *p* > 0.05). Doses of 4,100–7,500 IU/day achieved the greatest reduction in insulin resistance (*p* < 0.01) without significantly affecting HbA1c (*p* > 0.05). With respect to exercise interventions, intensities ≤4 MET significantly improved insulin resistance and insulin levels (both *p* < 0.01) and significantly affected HbA1c (*p* < 0.01) and FBS (*p* < 0.05). Intensities of 4.1–8 MET optimized HbA1c outcomes (*p* < 0.01) but did not significantly affect FINS levels (*p* > 0.05). Intensities of 8.1–15 MET significantly improved HbA1c and FBS (both *p* < 0.01) but did not significantly influence IR or FINS (both *p* > 0.05). An intervention frequency of three sessions per week significantly improved IR, HbA1c, and FINS outcomes. Single-session durations ≤60 min produced the greatest improvements in IR and FINS levels, whereas durations ≤45 min were most effective for HbA1c and FBS.

On the basis of these findings, fat-soluble vitamin supplementation at 4,100–7,500 IU/day combined with exercise at approximately 4 METs, performed three times per week, with each session lasting 45–60 min, is recommended to achieve optimal improvements in insulin resistance and related metabolic indicators in patients with type 2 diabetes. The detailed results are presented in Tables 3–5.

**Table 3:** Subgroup reactions of vitamin supplementation to insulin resistance and corresponding indicator doses.

Covariates and stratified subgroups	Number of studies	Effect size (confidence interval)	*P*	Heterogeneity	*P*
				I^2^/%	
Insulin resistance					
0–2000	6	−0.906 (−1.651, −0.160)	0.017.	90.1	0.000
2,100–4,000	3	−0.215 (−0.786, 0.355)	0.459	39.6	0.191
4,100–7,500	5	−0.789 (−1.249, −0.330)	0.001	63.2	0.0028
Glycated hemoglobin					
0–2000	6	−0.638 (−1.339, 0.064)	0.075	87.9	0.000
2,100–4,000	1	−1.397 (−2.297, −0.496)	0.002	–	–
4,100–7,500	4	−0.694 (−1.500, 0.113)	0.092	80.8	0.001
Fasting blood glucose					
0–2000	4	−1.093 (−1.767, −0.419)	0.001	77.6	0.004
2,100–4,000	2	−0.303 (−0.898, −0.292)	0.318	0.0	0.978
4,100–7,500	4	−0.810 (−1.477, −0.144)	0.017	89.8	0.000
Insulin					
0–2000	4	−0.399 (−0.697, 1.496)	0.475	87.5	0.000
2,100–4,000	3	−0.425 (−0.751, −0.100)	0.010	0.0	0.923
4,100–7,500	4	−0.978 (−1.868, −0.088)	0.031	83.4	0.000

**Table 4:** Dose response of exercise intensity to insulin resistance and corresponding indicators.

Covariates and stratified subgroups	Number of studies	Effect size (confidence interval)	*P*	Heterogeneity	*P*
				I^2^/%	
Insulin resistance					
0–4	9	−1.108 (−1.349, −0.630)	0.000	87.4	0.000
4.1–8	8	−0.742 (−1.211, −0.273)	0.002	69.9	0.002
8.1–15	6	−1.034 (−1.970, −0.099)	0.030	93.6	0.000
Glycated hemoglobin					
0–4	4	−1.066 (−2.046, −0.086)	0.033	84.1	0.000
4.1–8	8	−0.945 (−1.409, −0.482)	0.000	77.1.0	0.000
8.1–15	4	−1.095 (−1.549, −0.640)	0.000	53.9	0.089
Fasting blood glucose					
0–4	6	−2.107 (−3.483, −0.731)	0.003	97.0	0.000
4.1–8	8	−0.776 (−1.430, −0.121)	0.020	87.8	0.000
8.1–15	5	−0.967 (−1.404, −0.530)	0.000	72.7	0.005
Insulin					
0–4	9	−0.940 (−1.453, −0.427)	0.000	82.5	0.000
4.1–8	5	−0.905 (−2.177, 0.367)	0.163	89.2	0.000
8.1–15	5	−0.359 (−0.999, 0.280)	0.271	83.0	0.000

**Table 5:** Subgroup analysis of exercise intervention frequency and duration of single exercise intervention.

Index	Compare types	Effect value	*P*
Insulin resistance	3 times/week	−1.059 (−1.700, −0.418)	0.001
	>3 times/week	−0.455 (−1.028, 0.119)	0.120
	45 min	−0.983 (−1.941, −0.024)	0.044
	60 min	−0.934 (−1.729, −0.139)	0.021
	30 min	−0.599 (−1.803, 0.605)	0.330
	Total	−0.868 (−1.364, −0.372)	0.001
Glycosylated hemoglobin	3 times/week	−1.068 (−1.495, −0.641)	0.000
	>3 times/week	−0.683 (−1.134, −0.231)	0.003
	45 min	−1.105 (−1.507, −0.703)	0.000
	60 min	−0.906 (−1.466, −0.345)	0.002
	30 min	−0.100 (−0.777, 0.577)	0.772
	total	−0.955 (−1.285, −0.624)	0.000
Fasting blood sugar	3 times/week	−0.270 (−0.926, 0.386)	0.419
	>3 times/week	0.053 (−0.945, 1.052)	0.916
	45 min	−1.108 (−1.838, −0.379)	0.003
	60 min	0.933 (−0.063, 1.928)	0.066
	30 min	−0.730 (−1.318, −0.143)	0.015
	total	−0.214 (−0.746, 0.318)	0.430
Insulin	3 times/week	−1.133 (−1.856, −0.410)	0.002
	>3 times/week	−0.144 (−1.471, 1.036)	0.811
	45 min	−0.878 (−1.814, 0.059)	0.066
	60 min	−1.375 (−2.731, −0.020)	0.047
	30 min	−0.525 (−2.005, 0.954)	0.486
	total	−0.896 (−1.471, −0.320)	0.002

### Network meta-analysis results

#### Network plot

**Figure 3:**
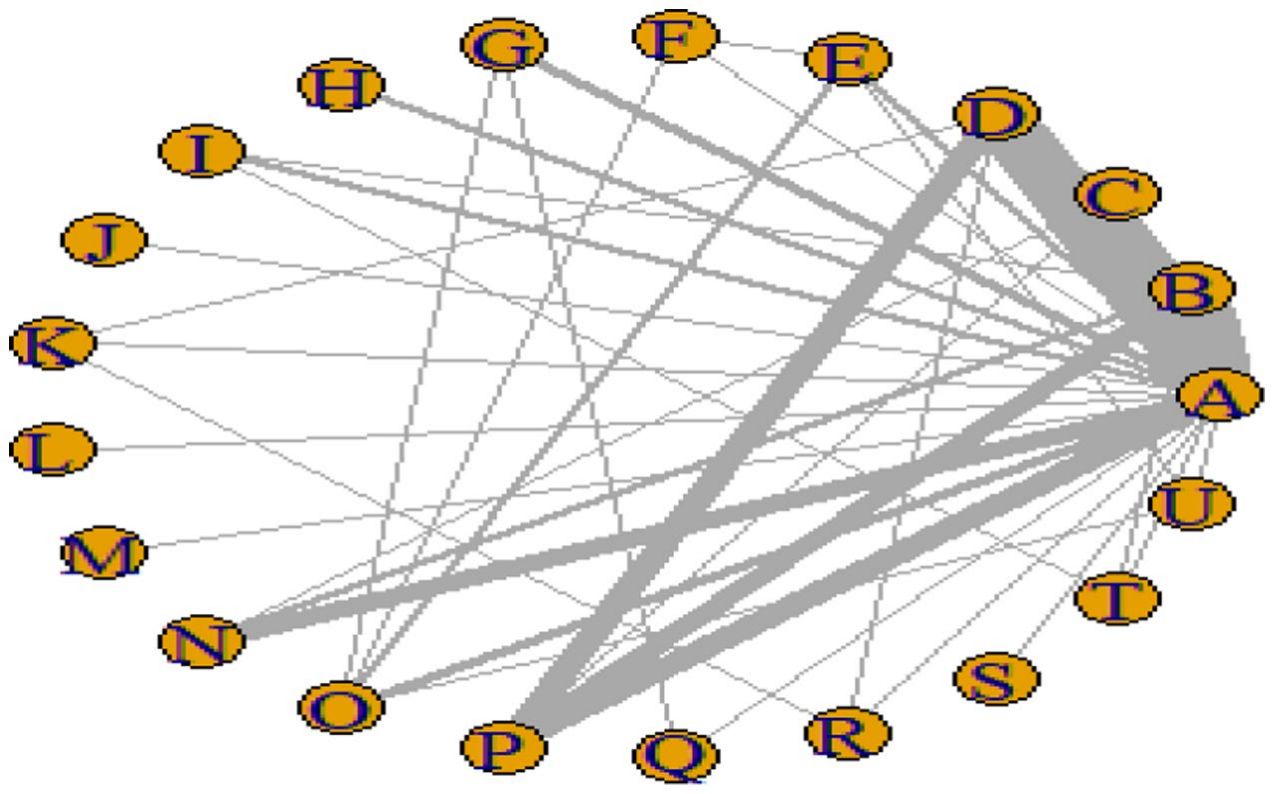
Network diagram of insulin resistance outcome indicators.

**Figure 4:**
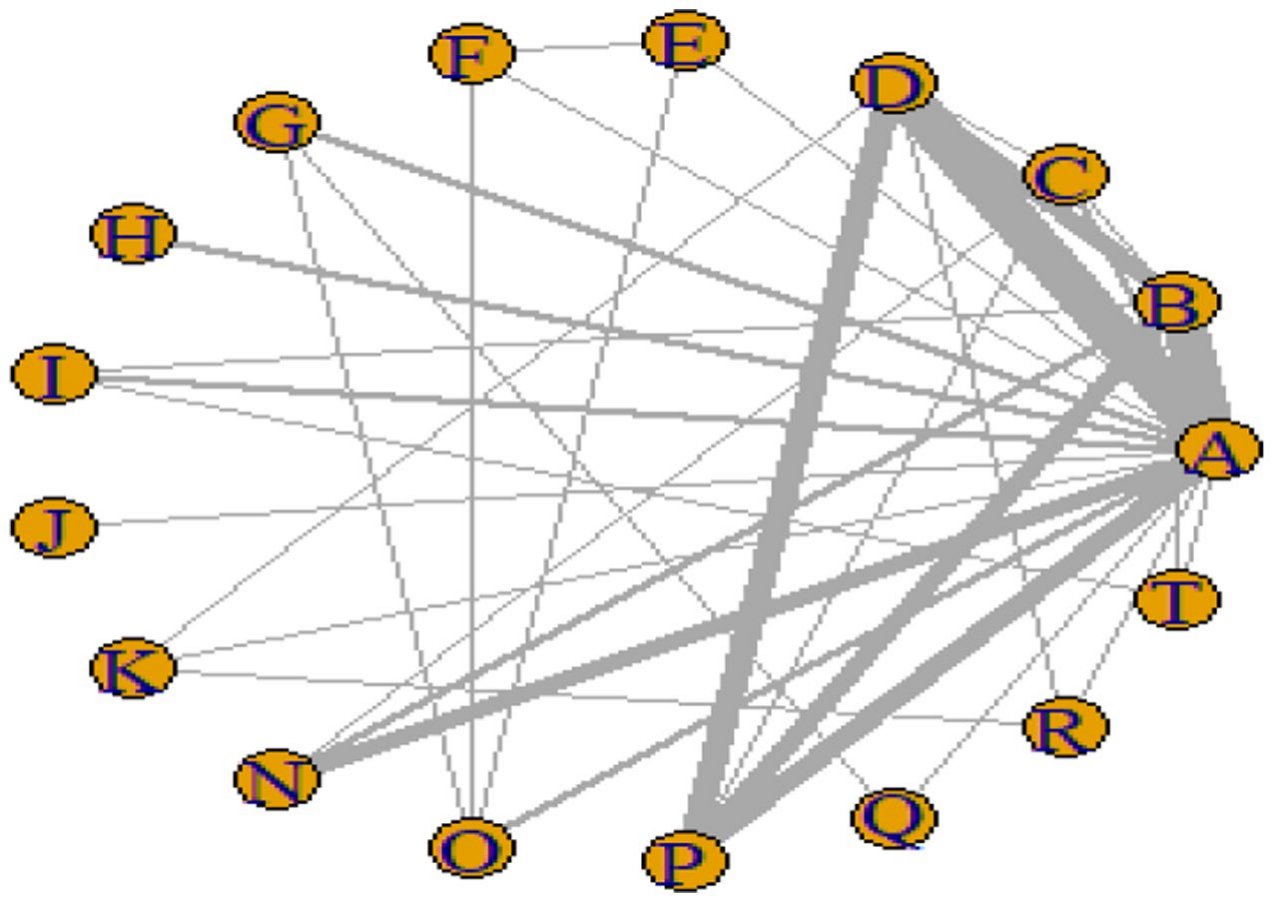
Network diagram of glycated hemoglobin outcome indicators.

**Figure 5:**
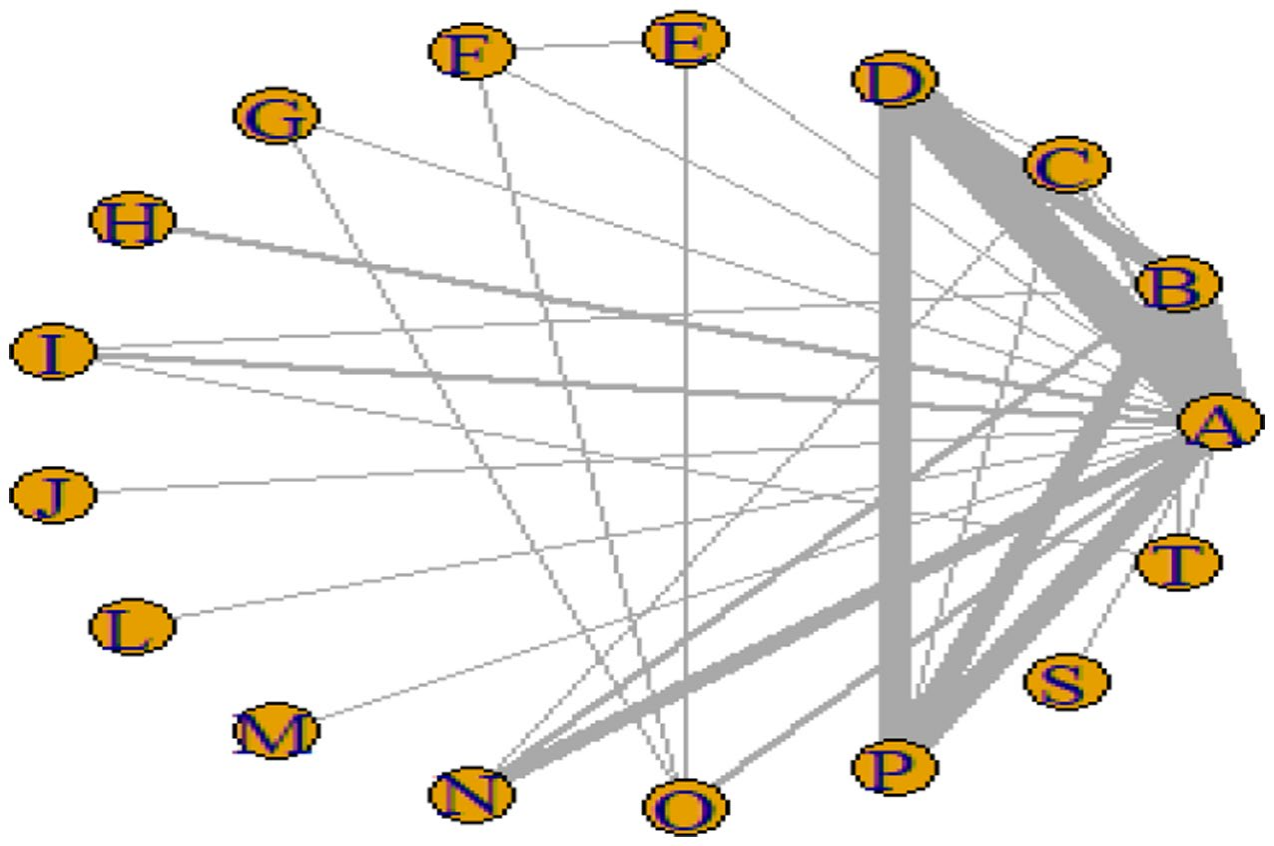
Network diagram of fasting blood glucose outcome indicators.

**Figure 6:**
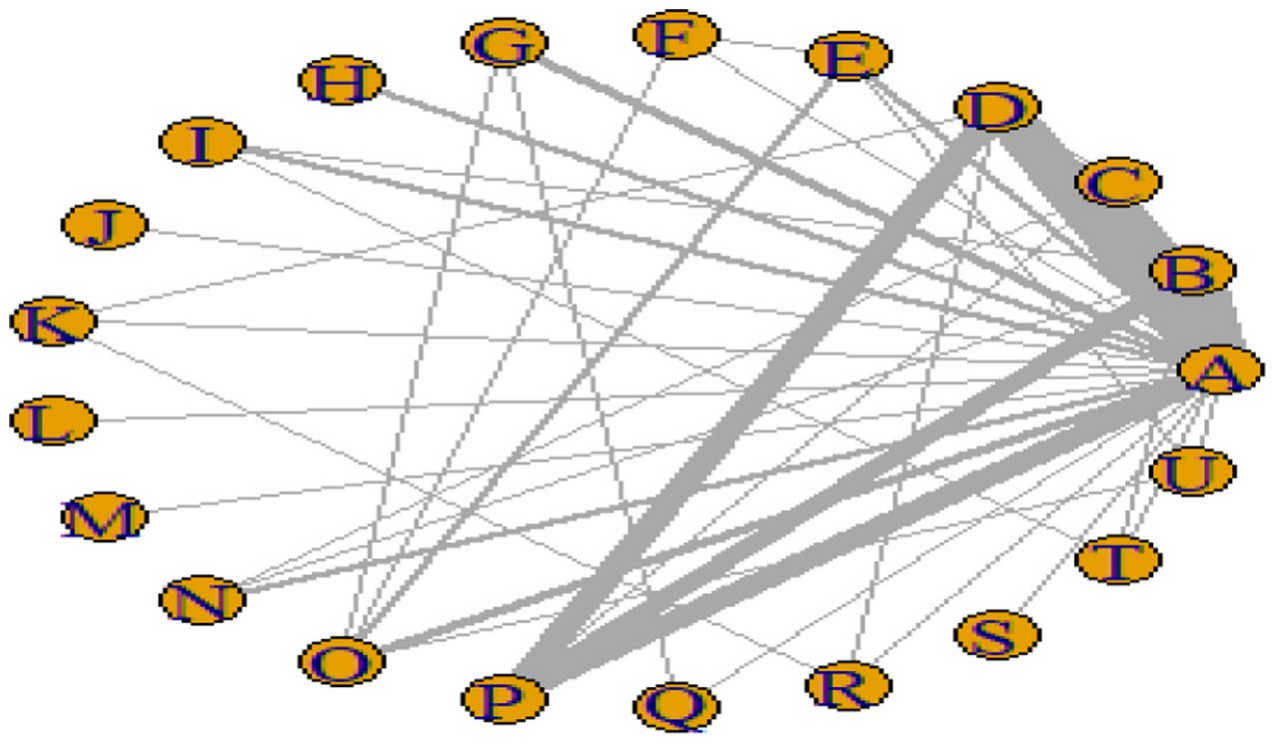
Network diagram of insulin outcome indicators.

Figures 3–6 illustrate the network relationships of various interventions—including vitamin supplementation, unsaturated fatty acids, trace elements, curcumin, aerobic exercise, resistance training, combined vitamin and exercise interventions, and combinations of vitamins with unsaturated fatty acids plus exercise—on insulin resistance and related metabolic indicators (glucose, insulin, and hemoglobin A1c) in patients with type 2 diabetes. All the network plots were centered on the control group and did not form closed loops. Overall, the comparisons focused primarily on aerobic exercise, resistance training, vitamin D supplementation, and combined vitamin plus exercise interventions relative to the control group outcomes.

#### Forest plot

**Figure 7:**
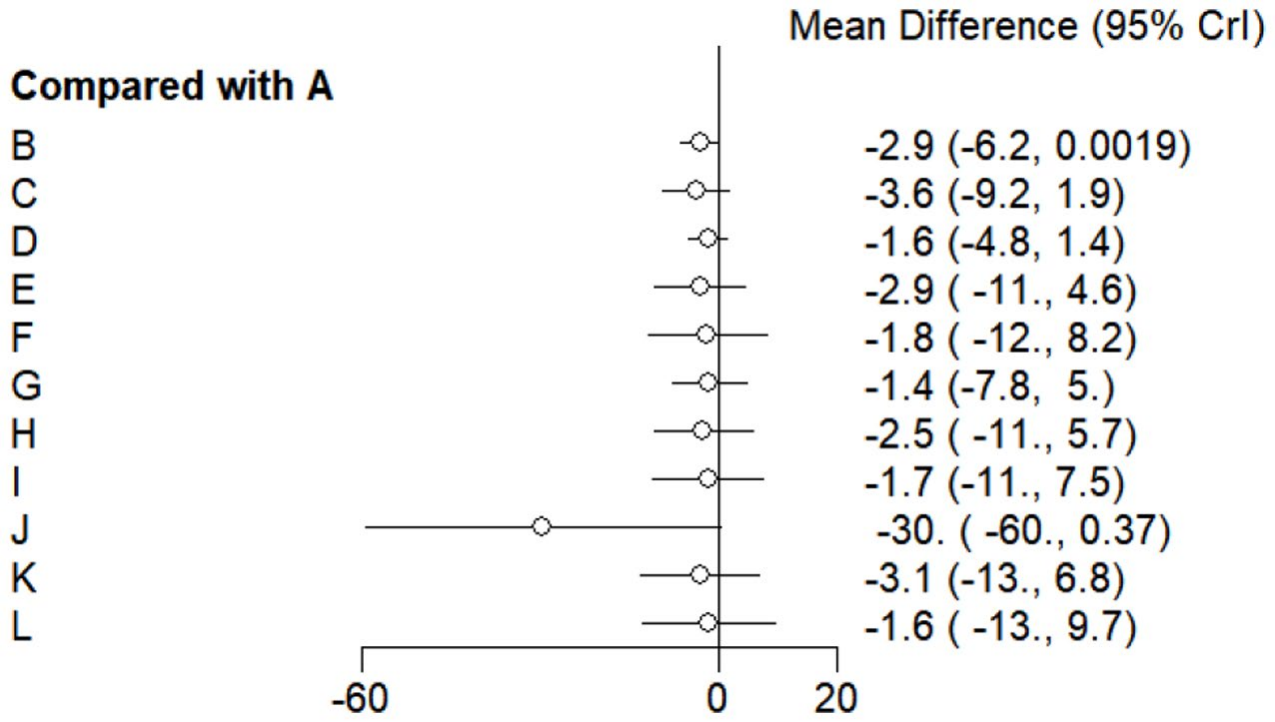
Forest plot of insulin resistance outcome indicators.

**Figure 8:**
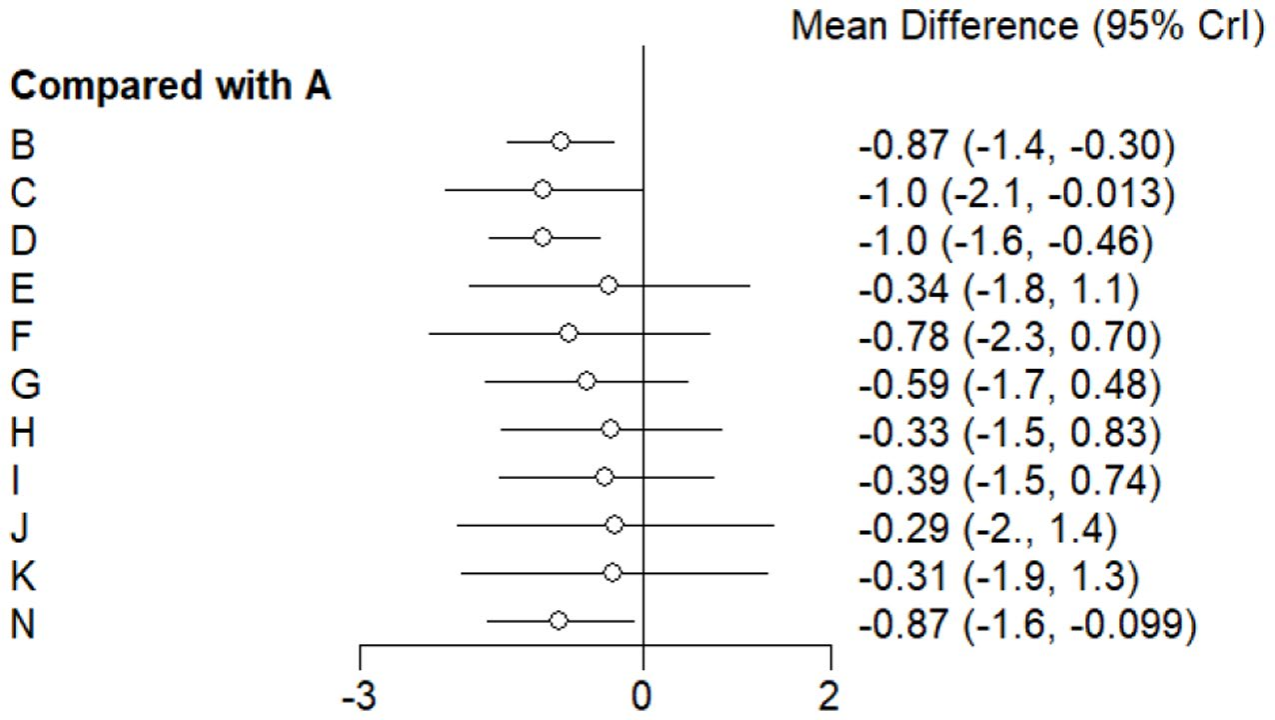
Forest plot of glycated hemoglobin outcome indicators.

**Figure 9:**
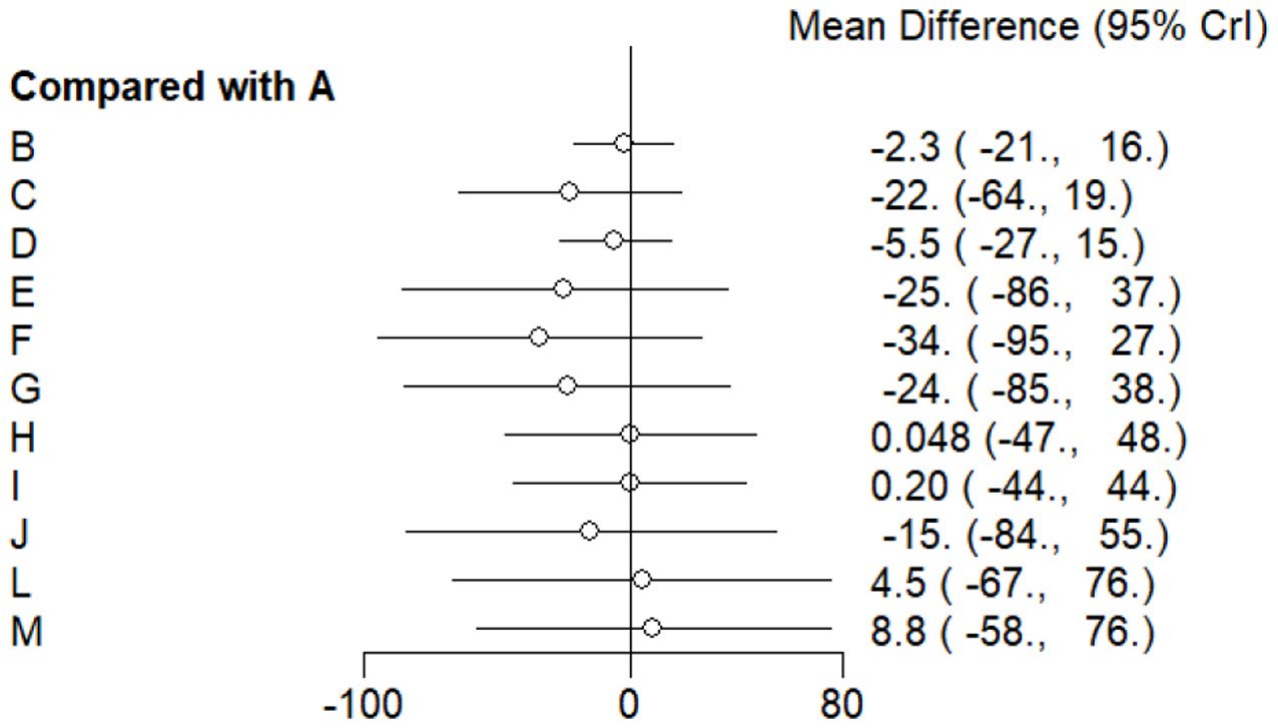
Forest plot of fasting blood glucose outcome indicators.

**Figure 10:**
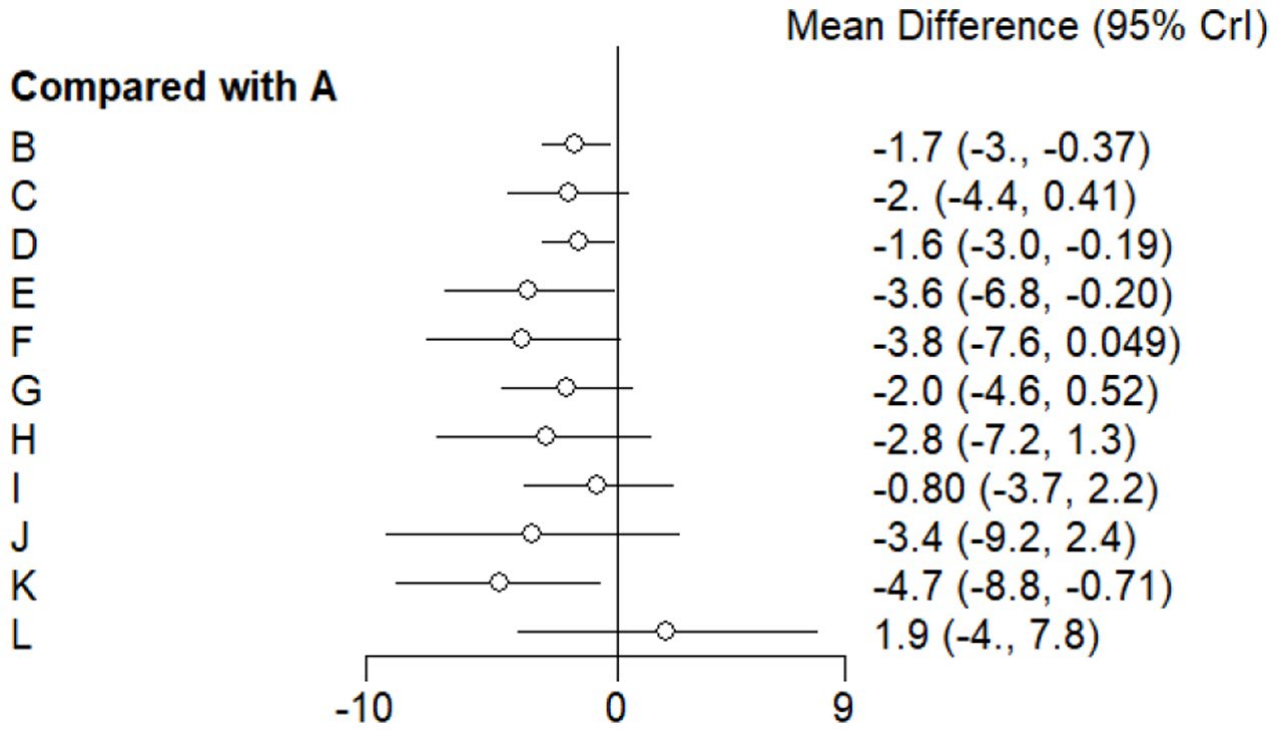
Forest plot of insulin outcome indicators.

This study included 33 trials on single vitamin supplementation, 21 trials on resistance training, 36 trials on aerobic exercise, and 28 trials on combined interventions, including vitamin plus exercise, combined vitamin supplementation, combined exercise, exercise plus probiotics, and exercise plus unsaturated fatty acids. The results of the network meta-analysis indicated that, compared with the control diet without exercise or corresponding vitamin interventions, probiotics produced the greatest improvement in insulin resistance. Vitamin D significantly improved HbA1c, whereas vitamin C had the most pronounced effects on FBS and insulin levels. The detailed results are presented in Figures 7–10.

#### Consistency test and model convergence diagnosis

In this study, trace plots were used to assess model convergence, whereas bandwidth indicators from density plots were used to evaluate model stability. Theoretically, as the number of iterations increases sufficiently, a bandwidth approaching zero indicates progressively enhanced model stability. The results revealed that insulin resistance values ranged from 0.08804 to 0.5455, with the highest value observed for d.A.S. (0.5455). HbA1c ranged from 0.4257 to 3.512, with a d.A.L. of 3.512 as the peak value. Insulin values ranged from 0.06296 to 0.2959, peaking at d.A.M. (0.2959), and FBS ranged from 0.4257 to 3.512, also peaking at d.A.L (3.512). Most sd.d values were low, whereas d.A.L represented a core high value across indicators. Overall, HbA1c and FBS exhibited greater density and more complete information (see Table 6).

**Table 6:** Inclusion of study trajectory density information.

Index	Compare types	Bandwidth
Insulin resistance	Density of d.A.B	0.1498
	Density of d.A.C	0.2709
	Density of d.A.D	0.1507
	Density of d.A.E	0.3672
	Density of d.A.F	0.4794
	Density of d.A.G	0.3052
	Density of d.A.H	0.3952
	Density of d.A.I	0.447
	Density of d.A.J	0.1483
	Density of d.A.K	0.48
	Density of d.A.L	0.5443
	Density of d.A.M	0.5435
	Density of d.A.N	0.2284
	Density of d.A.O	0.3037
	Density of d.A.P	0.1962
	Density of d.A.Q	0.4927
	Density of d.A.R	0.479
	Density of d.A.S	0.5455
	Density of d.A.T	0.4794
	Density of d.A.U	0.4929
	Density of sd.d	0.08804
Glycosylated hemoglobin	Density of d.A.B	0.8906
	Density of d.A.C	2.052
	Density of d.A.D	1.033
	Density of d.A.E	2.971
	Density of d.A.F	2.962
	Density of d.A.G	2.969
	Density of d.A.H	2.301
	Density of d.A.I	2.139
	Density of d.A.J	3.409
	Density of d.A.L	3.512
	Density of d.A.M	3.265
	Density of d.A.N	1.505
	Density of sd.d	0.4257
Insulin	Density of d.A.B	0.06296
	Density of d.A.C	0.1177
	Density of d.A.D	0.06826
	Density of d.A.E	0.1613
	Density of d.A.F	0.1845
	Density of d.A.G	0.1234
	Density of d.A.H	0.2068
	Density of d.A.I	0.1412
	Density of d.A.J	0.2884
	Density of d.A.K	0.1979
	Density of d.A.L	0.2945
	Density of d.A.M	0.2959
	Density of d.A.N	0.1466
	Density of d.A.O	0.1252
	Density of d.A.P	0.07999
	Density of d.A.Q	0.1897
Fasting blood sugar	Density of d.A.B	0.8906
	Density of d.A.C	2.052
	Density of d.A.D	1.033
	Density of d.A.E	2.971
	Density of d.A.F	2.962
	Density of d.A.G	2.969
	Density of d.A.H	2.301
	Density of d.A.I	2.139
	Density of d.A.J	3.409
	Density of d.A.L	3.512
	Density of d.A.M	3.165
	Density of d.A.N	1.505
	Density of d.A.O	2.258
	Density of d.A.P	1.19
	Density of d.A.S	3.347
	Density of d.A.T	2.709
	Density of sd.d	0.4257

This study employed the node-splitting method to evaluate consistency among interventions for IR, HbA1c, FBS, and FINS outcomes. The analysis indicated that insulin resistance outcomes for interventions B versus N, as well as insulin outcomes for interventions B versus N, demonstrated poor consistency (*p* < 0.05). All the other outcome measures showed good consistency across the interventions (*p* > 0.05). The detailed results are presented in Table 7.

**Table 7:** Consistency test of insulin resistance and corresponding indicators.

Index	Compare types	*P*
Insulin resistance	d.B.C	0.099550
	d.B.D	0.473625
	d.B.I	0.984200
	d.B.N	0.023125
	d.B.P	0.643725
	d.C.D	0.717500
	d.C.N	0.781750
	d.C.P	0.776525
Glycosylated hemoglobin	d.B.C	0.565375
	d.B.D	0.567775
	d.B.I	0.738125
	d.B.N	0.206625
	d.B.P	0.736200
	d.C.D	0.640075
	d.C.N	0.341800
	d.C.P	0.471850
	d.G.O	0.714450
Fasting blood sugar	d.B.C	0.688525
	d.B.D	0.879550
	d.B.I	0.975625
	d.B.N	0.766325
	d.B.P	0.554425
	d.C.D	0.749575
	d.C.N	0.641250
	d.C.P	0.900200
Insulin	d.B.C	0.251450
	d.B.D	0.394850
	d.B.I	0.678950
	d.B.N	0.008875
	d.B.P	0.916325
	d.C.D	0.898250
	d.C.N	0.133575
	d.C.P	0.885075
	d.G.O	0.196125

### Cumulative ranking results

This study ranked the effects of vitamin supplementation, exercise interventions, and combined vitamin plus exercise interventions on IR and related metabolic outcomes (FBS, FINS, and HbA1c) in patients with type 2 diabetes via SUCRA values. The SUCRA values range from 0 to 1, with values approaching 1 indicating maximal efficacy and values near 0 indicating minimal efficacy. The ranking results demonstrated that probiotics produced the greatest improvement in insulin resistance. Combined vitamin plus exercise interventions achieved the most pronounced effect on hemoglobin A1c. Vitamin C supplementation significantly reduced FBS levels. Combined vitamin plus exercise interventions again had the optimal effect on hemoglobin A1c outcomes, whereas fatty acid plus exercise interventions produced the greatest improvement in insulin levels. The detailed results are presented in Table 8 and Appendix 1.

**Table 8:** Cumulative probability results (SUCRA values) of network meta-analysis on insulin resistance and corresponding indicators under different intervention methods.

Intervention measures	Insulin resistance	Fasting blood sugar	Insulin	Glycosylated hemoglobin
A	0.262	0.309	0.140	0.164
B	0.574	0.356	0.376	0.610
C	0.611	0.643	0.430	0.679
D	0.438	0.415	0.357	0.706
E	0.551	0.636	0.648	0.365
F	0.463	0.729	0.671	0.553
G	0.427	0.625	0.436	0.466
H	0.509	0.363	0.552	0.350
I	0.455	0.356	0.270	0.370
J	0.959	0.534	0.604	0.358
K	0.541	*	0.770	0.363
L	0.454	0.350	0.124	*
M	0.323	0.304	0.094	*
N	0.523	0.561	0.348	0.608
O	0.576	0.700	0.794	0.480
P	0.538	0.593	0.539	0.968
Q	0.442	*	0.822	0.510
R	0.521	*	0.733	0.328
S	0.401	0.479	0.496	*
T	0.476	0.547	0.747	0.622
U	0.451	*	0.547	*

*: None.

### Sensitivity analysis

**Figure 11:**
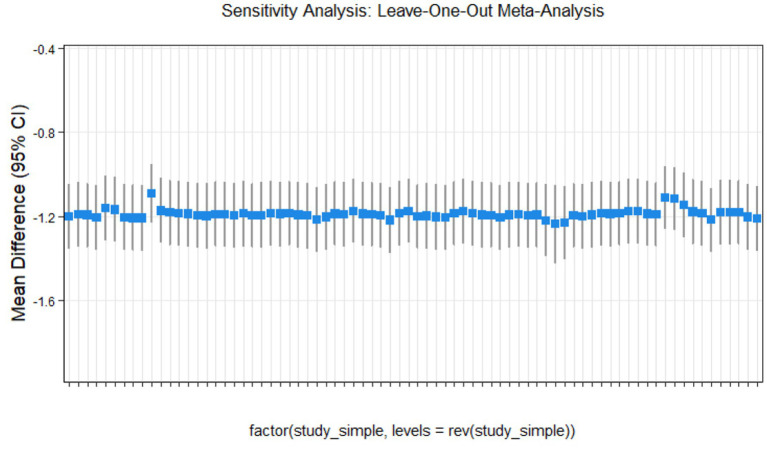
Sensitivity analysis of insulin resistance outcome indicators.

**Figure 12:**
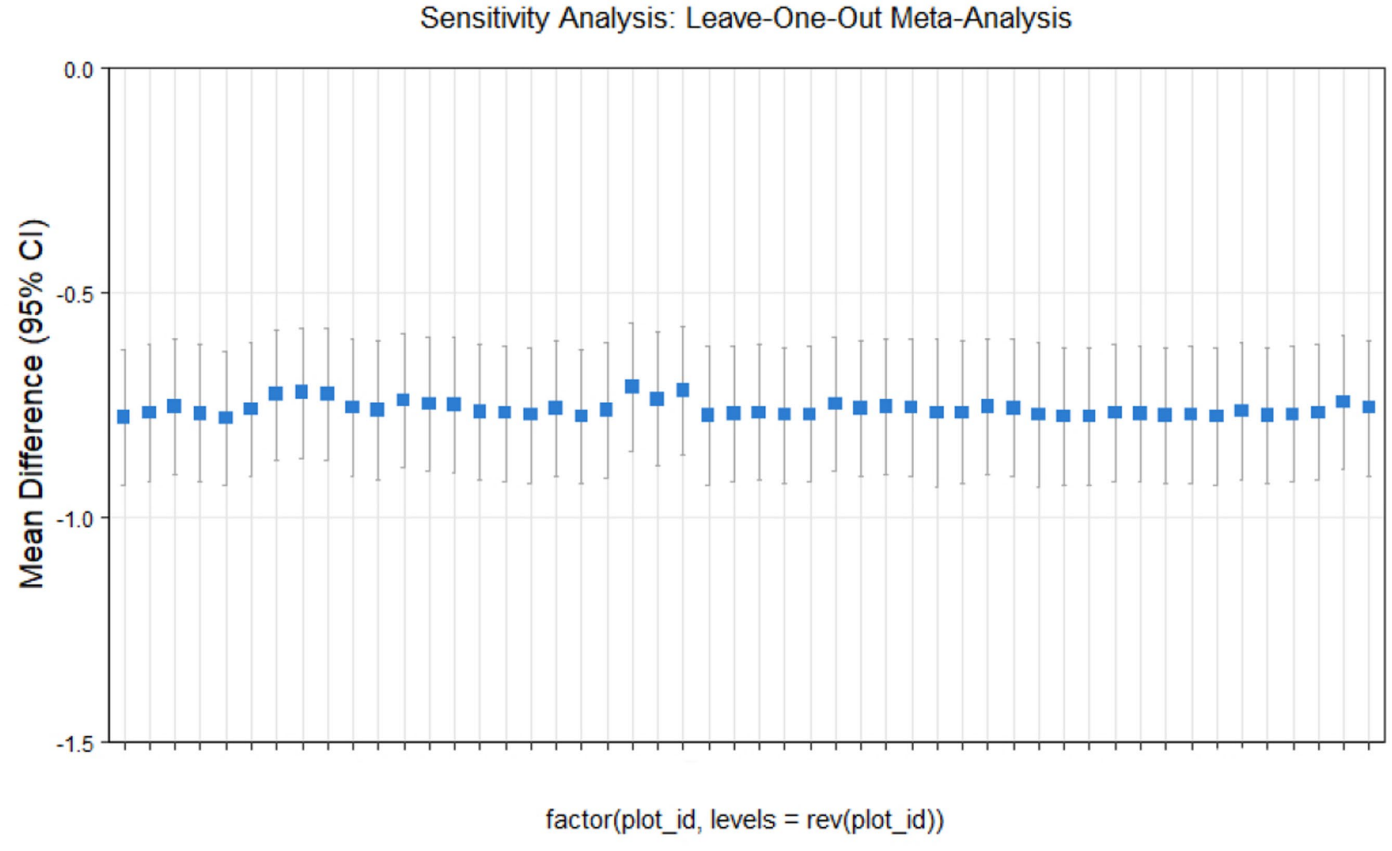
Sensitivity analysis of glycated hemoglobin outcome indicators.

**Figure 13:**
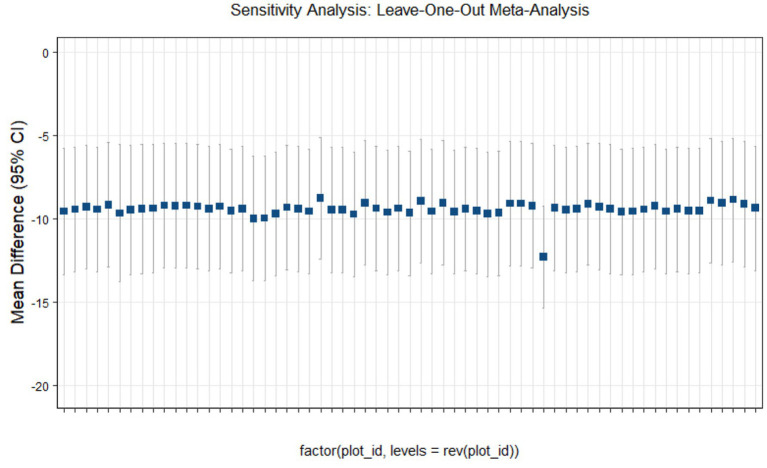
Sensitivity analysis of fasting blood glucose outcome indicators.

**Figure 14:**
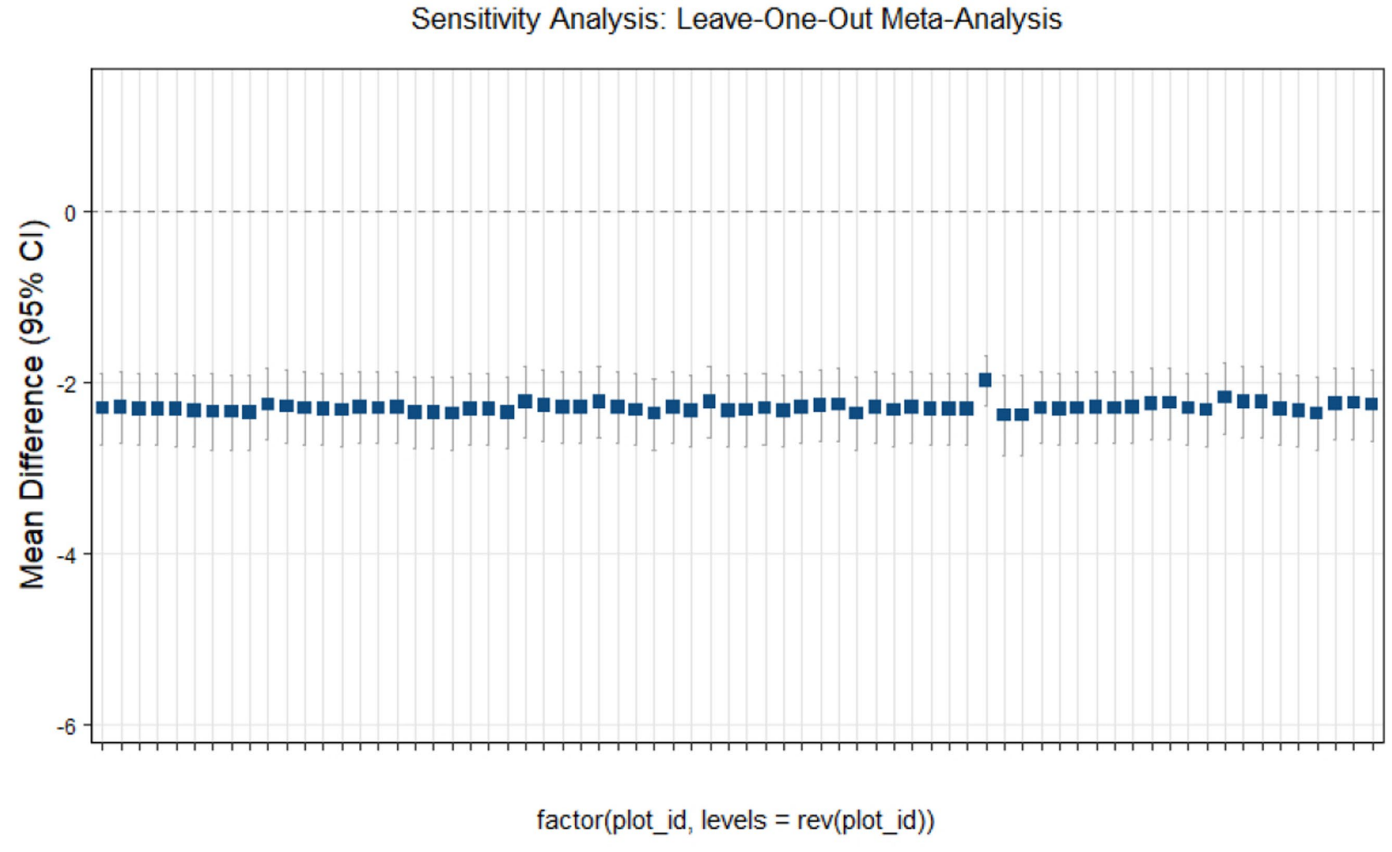
Sensitivity analysis of insulin outcome indicators.

This study conducted sensitivity analyses via R software, as shown in Figures 11–14. The meta-analysis results for IR, HbA1c, FBS, and FINS outcomes remained stable, with no evidence of significant heterogeneity detected.

### Heterogeneity test

This study used Egger’s and Begg’s tests to assess heterogeneity among the included outcome measures. The results indicated significant heterogeneity for IR, HbA1c, FBS, and FINS outcomes. The detailed results are presented in Table 9.

**Table 9:** Heterogeneity test table for insulin resistance and corresponding indicators.

Outcome indicators	Egger’s test (P)	Begg’s test (P)
Insulin resistance	0.000	0.000
Glycosylated hemoglobin	0.030	0.001
Fasting blood sugar	0.010	0.000
Insulin	0.000	0.000

### Regression analysis

This study employed Stata software to perform meta-regression analyses to identify sources of heterogeneity among the included studies. We treated the pooled effect sizes as the dependent variable and used intervention duration and frequency as covariates to construct the meta-regression model. The results indicated that combined exercise and vitamin interventions did not reveal significant sources of heterogeneity for IR, FBS, or FINS outcomes (*p* > 0.05). Similarly, intervention duration and frequency did not account for heterogeneity in hemoglobin A1c outcomes (*p* > 0.05). However, heterogeneity in HbA1c outcomes was detected (*p* < 0.05). The detailed results are presented in Table 10.

**Table 10:** Regression analysis of insulin resistance and corresponding indicators.

Outcome indicators	_ES	(95% Conf. Interval)	*T*	*P*
Insulin resistance	Week	(−1.189681, 0.1976864)	−1.43	0.158
	Methods	(−0.7155258, 0.1739014)	−1.22	0.228
	–	(−1.593843, 1.947468)	0.20	0.842
Glycosylated hemoglobin	Week	(−0.0714416, 0.7453682)	1.67	0.103
	Methods	(−0.2460263, 0.2547492)	0.04	0.972
	–	(−2.940112, −0.669356)	−3.21	0.003
Fasting blood sugar	Week	(−0.2485223, 1.737867)	1.51	0.138
	Methods	(−0.8904513, 0.1703226)	−1.37	0.178
	–	(−4.500902, 0.4960883)	−1.61	0.113
Insulin	Week	(−0.2428625, 1.225148)	1.34	0.185
	Methods	(−0.682556, 0.1502671)	−1.28	0.205
	–	(−3.210808, 0.2367608)	−1.73	0.089

### Evaluation of evidence quality

**Figure 15:**
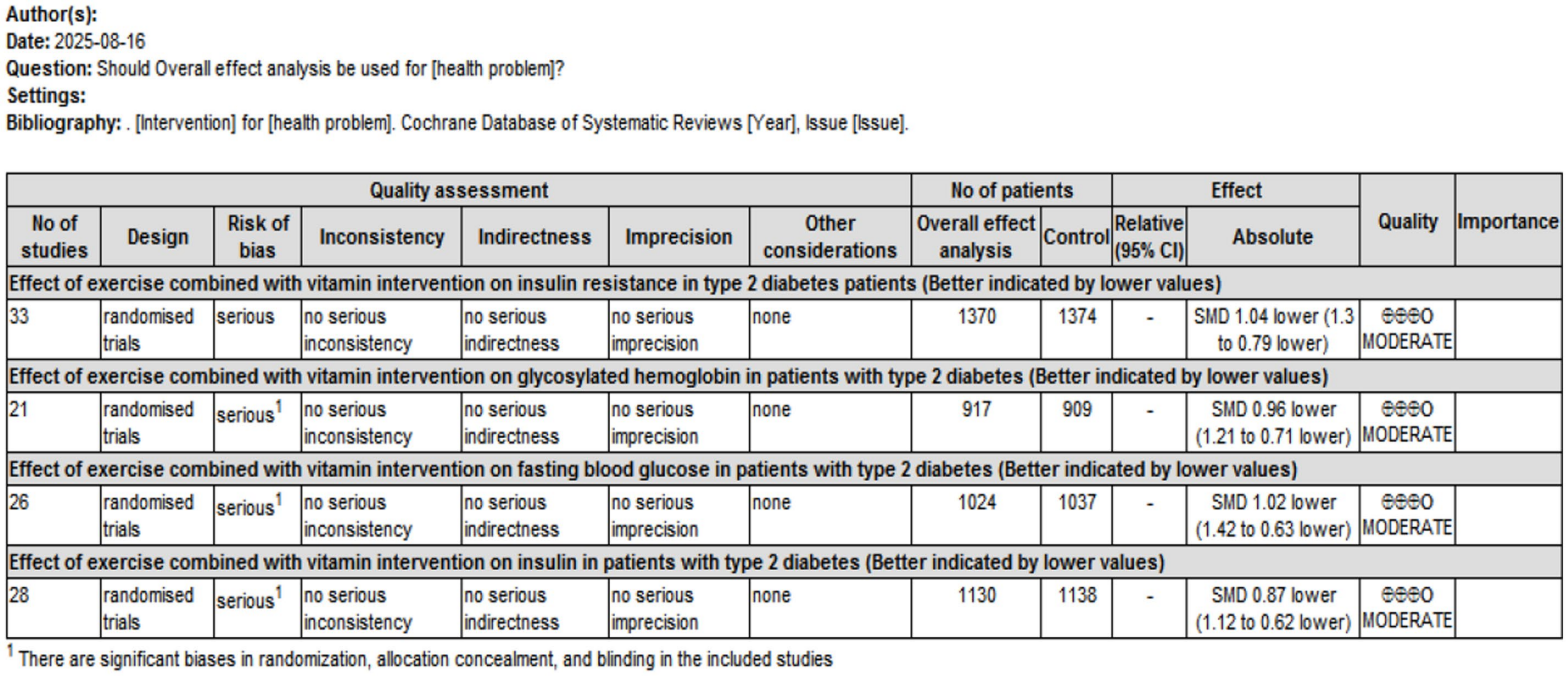
Quality of evidence for insulin resistance and corresponding outcome indicators.

This study used the GRADE framework to evaluate the quality of the included outcome measures. The assessment indicated that IR, HbA1c, FBS, and FINS outcomes were moderate. The detailed results are presented in Figure 15.

## Discussion

This study employed network meta-analysis to evaluate the effects of combined exercise and vitamin interventions on insulin resistance and related metabolic indicators in patients with T2D. The traditional meta-analysis results indicated that intervention periods of both <12 weeks and >12 weeks significantly improved insulin resistance and related indicators. Except for the effects of combined vitamin interventions on HbA1c, single exercise or single vitamin interventions on fasting blood glucose, and combined exercise interventions on FINS levels, all other intervention strategies produced significant improvements in IR and associated metrics. Dose–response subgroup analysis revealed that vitamin supplementation at 4,100–7,500 IU/day combined with exercise at approximately 4 MET, performed three times per week with each session lasting 45–60 min, produced optimal improvements in insulin resistance and related metabolic indicators.

Network meta-analysis further demonstrated that probiotic supplementation most effectively improved insulin resistance in T2D patients; combined exercise and vitamin interventions had the greatest effect on HbA1c; vitamin C supplementation most significantly improved FBS and insulin levels; and combined fatty acid and exercise interventions had the strongest effect on FINS levels. Specifically, interventions with periods >12 weeks or <12 weeks, exercise performed three times per week, and single-session durations of 45–60 min at ~4 MET, combined with weekly vitamin supplementation of 5,000 IU achieved the most pronounced effects on IR and related outcomes. These findings provide evidence-based guidance for designing exercise plus vitamin intervention programs for T2D patients.

The results highlight the importance of specific intervention doses and intensities. Exercise at ≤4 MET significantly improved insulin resistance and insulin levels (*p* < 0.01) and significantly modulated HbA1c (*p* < 0.01) and FBS (*p* < 0.05). Vitamin supplementation at 4,100–7,500 IU/day significantly affected all measured outcomes except HbA1c. Furthermore, combining exercise with vitamin and nutrient supplementation produced even greater improvements in IR and related indicators. Exercise not only reduces body weight but also enhances glycemic control, decreases body fat, and improves insulin responsiveness. Vitamins reduce oxidative stress by scavenging reactive oxygen species (ROS) and reactive nitrogen species (RNS), thereby improving insulin signaling pathways.

Insulin serves as a central regulator of metabolic homeostasis, and dysregulation of insulin secretion and action is closely linked to modern sedentary lifestyles, overnutrition, and metabolic syndrome, which are key contributors to T2D development. When pancreatic *β*-cells fail to compensate for IR, hyperglycemia occurs, promoting T2D progression. This process involves *β*-cell oxidative stress, inflammation, and disrupted redox signaling, which are often triggered by chronic nutrient overload leading to lipotoxicity and glucotoxicity (6). Vitamin D (VitD) exerts multiple metabolic regulatory effects via its nuclear receptor: it protects mitochondrial function by upregulating components of the respiratory chain, thereby reducing oxidative stress-mediated vascular complications; for example, 2,000 IU/day VitD supplementation for 3 months reduces DNA oxidative damage (7, 8). VitD also regulates *β*-cell Ca^2+^ flux as a chemical messenger, influencing insulin secretion by modulating extracellular Ca^2+^ concentration and membrane Ca^2+^ transport. VitD deficiency impairs *β*-cell secretory function, while supplementation (2,000–7,500 IU/day) restores intracellular Ca^2+^ levels and enhances insulin secretion (9). VitD deficiency also elevates parathyroid hormone (PTH) and induces mild pancreatic inflammation, both of which are related to IR (10). Supplementation increases anti-inflammatory cytokine IL-10 and decreases the proinflammatory cytokines TNF-*α*, IL-6, IL-12, and IFN-*γ*, thereby improving immune homeostasis and IR (11).

Clinical studies support these mechanisms. Twelve weeks of progressive endurance cycling (2–3 sessions/week, 1 h/session at 65–80% maximum heart rate) combined with 1,000 IU/day VitD3 did not improve HOMA-IR but significantly reduced FBS (11.9% change, effect size 0.65, *p* < 0.05) (12). Higher-dose interventions (50,000 IU of VitD weekly for 8 weeks) normalized serum 25-hydroxyvitamin D levels (*p* < 0.001) and significantly reduced FBS (*p* = 0.04), FINS (*p* = 0.02), and HOMA-IR (*p* = 0.007) (13). Another multi-arm study also demonstrated that 8 weeks of aerobic exercise (30 min, 50–75% heart rate reserve, 3 times per week) combined with 25 min of resistance training, along with daily supplementation of 4,000 IU of vitamin D₃, significantly reduced HOMA-IR [*F* = 24.07; *p* < 0.001], FINS levels [*F* = 17.97; *p* < 0.001], and FBS [*F* = 11.28; *p* < 0.001] compared to the placebo group. However, the combination of vitamin D₃ and exercise resulted in the highest percentage reductions (−20.73, −10.68%, −11.54%, respectively) (14). Furthermore, daily supplementation of 600 mg of calcium with 60 min of aerobic and resistance exercise also significantly reduced FBS, FINS, IR levels, and pro-inflammatory cytokines TNF-*α* and IL-6 (*p* < 0.005) compared to either exercise alone, vitamin D intervention, or the placebo group. The combination group had higher levels of 25(OH)D and fasting C-peptide than the vitamin D group, exercise group, and placebo group, and lower HbA1c levels than the other groups (*p* < 0.05). Additionally, the combination group exhibited lower levels of TNF-α and IL-6 compared to the vitamin D, exercise, and placebo groups, with statistically significant differences (*p* < 0.05) (15).

Exercise improves glucose uptake and metabolism through multiple pathways, including enhancing insulin sensitivity, promoting cellular glucose transport, and reducing fat accumulation, making it a critical intervention for T2D management (16). The regulation of blood glucose and fatty acid levels depends on energy utilization patterns: short-duration, high-intensity exercise relies primarily on glycogen, whereas long-duration, low-intensity exercise depends on fatty acid metabolism. Exercise intensity modulates glucose utilization via the dynamic balance between FINS and counterregulatory hormones such as glucagon, epinephrine, and cortisol (17, 18).

Clinical evidence shows that 12 weeks of low- to moderate-intensity running (40 min/session) significantly improved HOMA-IR (*F*(1,144) = 89.29, *p* < 0.001), fasting insulin (*F*(1,144) = 129.10, *p* < 0.001), FINS (*F*(1,144) = 12.193, *p* < 0.001), postprandial glucose (*F*(1,144) = 53.015, *p* < 0.001), and HbA1c (*F*(1,144) = 80.050, *p* < 0.001) (19). Twelve weeks of combined moderate-intensity walking (30 min) or high-intensity interval training (28 min) performed three times weekly also significantly improved fasting glucose, HbA1c, insulin, and HOMA-IR (20). Aerobic exercise performed three times per week for 25 weeks, totaling 75 min, or combined aerobic and resistance training, significantly reduced fasting blood glucose, improved glycemic control, and lowered plasma insulin and insulin resistance. However, the combined exercise group showed better outcomes in fasting blood glucose, HbA1c, insulin resistance index, peak exercise load, lean body mass, and overall quality of life scores compared to the aerobic-only group (*p* < 0.05) (21).

However, exercise effects vary. Ten weeks of resistance or endurance training (3 sessions/week, 40 min/session) significantly reduced insulin resistance but did not significantly improve FINS levels (*p* > 0.05) (21). Mechanistically, exercise enhances insulin-mediated glucose uptake in skeletal muscle, with resistance training resulting in particularly pronounced improvements, thereby reducing overall insulin dependence. A multiarm trial confirmed that 12 weeks of aerobic, resistance, or combined exercise (3 sessions/week, ~5.5 MET) reduced fasting glucose, but improvements in HOMA-IR were significant only in the aerobic and combined exercise groups (22).

Beyond metabolic regulation, exercise confers systemic benefits: it enhances cardiac function, lowers blood pressure, improves lipid profiles (reducing LDL-C and increasing HDL-C), and mitigates disease risk. Exercise also alleviates psychological stress, indirectly lowering glucose levels and reducing diabetes complications (18, 23). Studies have shown that exercise can improve HbA1c baseline levels by 10–20%, significantly optimizing T2D and insulin resistance status (24). This study also revealed marked improvements in HbA1c, which were further enhanced when vitamin supplementation was combined with vitamin C, suggesting a synergistic effect that warrants further investigation.

## Limitations and implications

This study represents the first effort to apply network meta-analysis and dose–response analysis to evaluate the effects of combined exercise and vitamin interventions on insulin resistance and related metabolic indicators in patients with type 2 diabetes (t2d). However, several limitations should be noted: 1. Limited standardization of outcomes: the included studies used inconsistent measurement units for key outcomes such as blood glucose (mg/dl vs. mmol/l), which may increase heterogeneity and reduce the precision of effect size estimates. 2. Subgroup analysis constraints: The analysis did not fully account for covariates such as sex, disease duration, or comorbidities (e.g., obesity, cardiovascular disease), limiting the ability to detect differential intervention effects across specific populations. 3. Intervention heterogeneity: differences in exercise modality (aerobic, resistance, or combined), intensity control, and vitamin supplementation (e.g., d3 vs. d2, timing of administration) may obscure the specificity of optimal intervention protocols. 4. Limited strength of evidence: direct comparison studies for some interventions, such as specific vitamin–plus–exercise combinations, are scarce. Network meta-analysis relies on indirect evidence to infer effects, which may reduce the robustness of conclusions. 5. Short follow-up periods: Most included trials had intervention durations ≤24 weeks, limiting the ability to assess long-term (≥1 year) effects on insulin resistance and diabetes-related complications.

Implications for future research: 1. To increase study design rigor, future large-scale, multidimensional RCTs should standardize outcome measurement and reporting, objectively monitor exercise intensity, evaluate vitamin bioavailability, and clearly document intervention details to minimize methodological heterogeneity. 2. Refined subgroup analyses: future studies should stratify t2d patients by sex, age, disease duration, and comorbidities to explore differential responses and support the development of individualized intervention strategies. 3. Precision intervention exploration: RCTs should investigate varying vitamin doses (e.g., 2000 iu vs. 4,100–7,500 iu), exercise intensities (4 met vs. higher), and combined intervention modes, using dose–response models to identify optimal thresholds. 4. Mechanistic investigation: Integrating molecular biology techniques, such as measuring inflammatory cytokines, oxidative stress markers, and gut microbiota composition, can clarify the biological mechanisms underlying exercise- and vitamin-mediated improvements in insulin resistance, including mitochondrial function regulation and the Ca^2+^ signaling pathway. 5. Long-term effect evaluation: Cohort studies with ≥1 year follow-up should examine sustained the impacts on diabetes and related complications, while evaluating adherence to interventions to provide comprehensive evidence for clinical practice. 6. Integrated multimodal interventions: Future research should explore combined strategies involving exercise, vitamins, dietary modifications, and psychological interventions, evaluating their synergistic effects on metabolic syndrome management and enhancing overall t2d prevention and control.

## Conclusion

Combined vitamin interventions produced the greatest improvements in FINS levels in patients with type 2 diabetes; vitamin C supplementation most effectively reduced FBS; Probiotic intervention has the best effect on IR robiotic intervention has the best effect on insulin resistance and intervention and combined vitamin-plus-exercise interventions achieved the optimal effect on HbA1c. Moreover, vitamin supplementation at 4,100–7,500 iu/day, exercise at approximately 4 met, and exercise three times per week for 45–60 min per session produced maximal improvements in insulin resistance and related metabolic indicators. Given the limitations of this study, high-quality experimental research is still needed to further validate these findings.

## Data Availability

The original contributions presented in the study are included in the article/Supplementary material, further inquiries can be directed to the corresponding author.

## References

[1] Zhang B. Effect of exercise on insulin resistance in obese type 2 diabetes patients. Rev Bras Med Esporte. (2022) 28:59–61. doi: 10.1590/1517-86922022v28n1p59-61

[2] Chen HJ Wang M Zou DM Liang GY Yang SY. Effects of vitamin family members on insulin resistance and diabetes complications. World J Diabetes. (2024) 15:568–71. doi: 10.4239/wjd.v15.i3.568, PMID: 38591081 PMC10999036

[3] Lee SH Park SY Choi CS. Insulin resistance: from mechanisms to therapeutic strategies. Diabetes Metab J. (2022) 46:15–37. doi: 10.4093/dmj.2021.0280, PMID: 34965646 PMC8831809

[4] Tambuwal UM Ahmad SA Hayatu U Sadiq MA Kolawale JA Bello SK . Exploring the effect of exercise versus metformin on insulin resistance amongst Nigerians with pre-diabetes: a randomised controlled trial. Niger Postgrad Med J. (2024) 31:274–9. doi: 10.4103/npmj.npmj_148_24, PMID: 39219352

[5] Rajabi-Naeeni M Dolatian M Qorbani M Vaezi AA. The effect of omega-3 and vitamin D co-supplementation on glycemic control and lipid profiles in reproductive-aged women with pre-diabetes and hypovitaminosis D: a randomized controlled trial. Diabetol Metab Syndr. (2020) 12:1–11. doi: 10.1186/s13098-020-00549-9, PMID: 32435279 PMC7218636

[6] Ježek P Jabůrek M Plecitá-Hlavatá L. Contribution of oxidative stress and impaired biogenesis of pancreatic β-cells to type 2 diabetes. Antioxid Redox Signal. (2019) 31:722–51. doi: 10.1089/ars.2018.7656, PMID: 30450940 PMC6708273

[7] Salum E Kals J Kampus P Salum T Zilmer K Aunapuu M . Vitamin D reduces deposition of advanced glycation end-products in the aortic wall and systemic oxidative stress in diabetic rats. Diabetes Res Clin Pract. (2013) 100:243–9. doi: 10.1016/j.diabres.2013.03.008, PMID: 23522919

[8] Wenclewska S Szymczak-Pajor I Drzewoski J Bunk M Śliwińska A. Vitamin D supplementation reduces both oxidative DNA damage and insulin resistance in the elderly with metabolic disorders. Int J Mol Sci. (2019) 20:2891. doi: 10.3390/ijms20122891, PMID: 31200560 PMC6628266

[9] Dos Santos GJ Ferreira SM Ortis F Rezende LF Li C Naji A . Metabolic memory of ß-cells controls insulin secretion and is mediated by CaMKII. Mol Metab. (2014) 3:484–9. doi: 10.1016/j.molmet.2014.03.011 24944908 PMC4060215

[10] Reis JP von Mühlen D Kritz-Silverstein D Wingard DL Barrett-Connor E. Vitamin D, parathyroid hormone levels, and the prevalence of metabolic syndrome in community-dwelling older adults. Diabetes Care. (2007) 30:1549–55. doi: 10.2337/dc06-2438, PMID: 17351276

[11] He LP Li CP Liu CW Gu W. The regulatory effect of vitamin D on pancreatic beta cell secretion in patients with type 2 diabetes. Curr Med Chem. (2025) 35:2890–8. doi: 10.2174/0109298673270429240805050928 39113297

[12] Sun X Yan T Li Z Zhou S Peng W Cui W . Effects of endurance exercise and vitamin D supplementation on insulin resistance and plasma Lipidome in middle-aged adults with type 2 diabetes. Nutrients. (2023) 15:3027. doi: 10.3390/nu15133027, PMID: 37447353 PMC10346630

[13] Baziar N Jafarian K Shadman Z Qorbani M Khoshniat Nikoo M Abd Mishani M. Effect of therapeutic dose of vitamin D on serum adiponectin and glycemia in vitamin D-insufficient or deficient type 2 diabetic patients. Iran Red Crescent Med J. (2014) 16:e21458. doi: 10.5812/ircmj.21458, PMID: 25593737 PMC4270651

[14] Rostamian Mashhadi M Bijeh N Rashidlamir A Raoof AA. Vitamin D3 supplementation could improve the effect of exercise training on type 2 diabetes-induced metabolic disorders via BDNF/irisin axis in elderly women. Sport Sci Health. (2024) 20:1281–90. doi: 10.1007/s11332-024-01204-w

[15] Yang SG Lin H Yang H. Effect of vitamin D combined with aerobic exercise on insulin resistance in elderly type 2 diabetic patients with vitamin D deficiency. Chin J Geriatr. (2020) 40:1893–6. doi: 10.3969/j.issn.1005-9202.2020.09.015

[16] Sigal RJ Kenny GP Wasserman DH Castaneda-Sceppa C White RD. Physical activity/exercise and type 2 diabetes: a consensus statement from the American Diabetes Association. Diabetes Care. (2006) 29:1433–8. doi: 10.2337/dc06-9910, PMID: 16732040

[17] Stokie JR Abbott G Howlett KF Hamilton DL Shaw CS. Intramuscular lipid utilization during exercise: a systematic review, meta-analysis, and meta-regression. J Appl Physiol. (2023) 134:581–92. doi: 10.1152/japplphysiol.00637.2021, PMID: 36656983

[18] Coyle EF. Substrate utilization during exercise in active people. Am J Clin Nutr. (1995) 61:968S–79S. doi: 10.1093/ajcn/61.4.968S, PMID: 7900696

[19] Amaravadi SK Maiya GA Vaishali K Shastry BA. Effectiveness of structured exercise program on insulin resistance and quality of life in type 2 diabetes mellitus–a randomized controlled trial. PLoS One. (2024) 19:e0302831. doi: 10.1371/journal.pone.0302831, PMID: 38771888 PMC11108169

[20] Martins FM de Paula Souza A Nunes PRP Michelin MA Murta EFC Resende EAMR . High-intensity body weight training is comparable to combined training in changes in muscle mass, physical performance, inflammatory markers and metabolic health in postmenopausal women at high risk for type 2 diabetes mellitus: a randomized controlled clinical trial. Exp Gerontol. (2018) 107:108–15. doi: 10.1016/j.exger.2018.02.016, PMID: 29471132

[21] Mogharnasi M TajiTabas A Tashakorizadeh M Nayebifar SH. The effects of resistance and endurance training on levels of nesfatin-1, HSP70, insulin resistance and body composition in women with type 2 diabetes mellitus. Sci Sports. (2019) 34:e15–23. doi: 10.1016/j.scispo.2018.04.010

[22] AminiLari Z Fararouei M Amanat S Sinaei E Dianatinasab S AminiLari M . The effect of 12 weeks aerobic, resistance, and combined exercises on omentin-1 levels and insulin resistance among type 2 diabetic middle-aged women. Diabetes Metab J. (2017) 41:205–12. doi: 10.4093/dmj.2017.41.3.205, PMID: 28537059 PMC5489501

[23] Piras A Raffi M. A narrative literature review on the role of exercise training in managing type 1 and type 2 diabetes mellitus. Healthcare. (2023) 11:2947. doi: 10.3390/healthcare11222947, PMID: 37998439 PMC10671220

[24] Gracia-Sánchez A López-Pineda A Chicharro-Luna E Gil-Guillén VF. A Delphi study protocol to identify recommendations on physical activity and exercise in patients with diabetes and risk of foot ulcerations. Int J Environ Res Public Health. (2021) 18:10988. doi: 10.3390/ijerph182010988, PMID: 34682736 PMC8536116

[25] Meng Q Chen W Zhang M Gao M. Effects of aerobic and resistance exercise combined on patients with type 2 diabetes. Chin J Rehabil Theory Pract. (2018) 24:1465–70. doi: 10.16386/j.cjpccd.issn.1006-9771.2018.12.011

[26] Imanparast F Javaheri J Kamankesh F Rafiei F Salehi A Mollaaliakbari Z . The effects of chromium and vitamin D3 co-supplementation on insulin resistance and tumor necrosis factor-alpha in type 2 diabetes: a randomized placebo-controlled trial. Appl Physiol Nutr Metab. (2020) 45:471–7. doi: 10.1139/apnm-2019-0113, PMID: 31593637

[27] Abd El-Aal A Abd El-Ghffar EA Ghali AA El-Aal AA El-Ghffar EAA Zughbur MR . The effect of vitamin C and/or E supplementation on type 2 diabetic adult males under metformin treatment: a single-blinded randomized controlled clinical trial. Diabetes Metab Syndr Clin Res Rev. (2018) 12:483–9. doi: 10.1016/j.dsx.2018.03.013, PMID: 29571976

[28] Aguayo-Ruiz JI García-Cobián TA Pascoe-González S Sánchez-Enríquez S Llamas-Covarrubias IM García-Iglesias T . Effect of supplementation with vitamins D3 and K2 on undercarboxylated osteocalcin and insulin serum levels in patients with type 2 diabetes mellitus: a randomized, double-blind, clinical trial. Diabetol Metab Syndr. (2020) 12:1–10. doi: 10.1186/s13098-020-00580-w 32831908 PMC7436967

[29] Raygan F Ostadmohammadi V Bahmani F Asemi Z. The effects of vitamin D and probiotic co-supplementation on mental health parameters and metabolic status in type 2 diabetic patients with coronary heart disease: a randomized, double-blind, placebo-controlled trial. Prog Neuro-Psychopharmacol Biol Psychiatry. (2018) 84:50–5. doi: 10.1016/j.pnpbp.2018.02.007, PMID: 29432877

[30] Darmian MA Hoseini R Amiri E Golshani S. How combined and separate aerobic training and turmeric supplementation alter lipid profile and glycemic status? A clinical trial in middle-aged females with type 2 diabetes and hyperlipidemia. Int Cardiovasc Res J. (2021) 15:120–27. doi: 10.15412/icrj2021v15n3p120-127

[31] de Oliveira AM Rondó PHC Luzia LA D’Abronzo FH Illison VK. The effects of lipoic acid and α-tocopherol supplementation on the lipid profile and insulin sensitivity of patients with type 2 diabetes mellitus: a randomized, double-blind, placebo-controlled trial. Diabetes Res Clin Pract. (2011) 92:253–60. doi: 10.1016/j.diabres.2011.02.010, PMID: 21371770

[32] Hua L Lei M Xue S Li X Li S Xie Q. Effect of fish oil supplementation combined with high-intensity interval training in newly diagnosed non-obese type 2 diabetes: a randomized controlled trial. J Clin Biochem Nutr. (2020) 66:146–51. doi: 10.3164/jcbn.19-64, PMID: 32231411 PMC7093295

[33] Yavari A Najafipoor F Aliasgarzadeh A Niafar M Mobasseri M. Effect of aerobic exercise, resistance training or combined training on glycaemic control and cardiovascular risk factors in patients with type 2 diabetes. Biol Sport. (2012) 29:135–43. doi: 10.5604/01.3001.0010.3934

[34] Ali AM Abbassi MM Sabry NA Fawzi M Mousa S. The effect of vitamin K4 supplementation on insulin resistance in individuals with type 2 diabetes: a double-blind randomised placebo-controlled clinical trial. Eur J Nutr. (2023) 62:3241–9. doi: 10.1007/s00394-023-03215-8, PMID: 37552330 PMC10611861

[35] Shabkhiz F Khalafi M Rosenkranz S Karimi P Moghadami K. Resistance training attenuates circulating FGF-21 and myostatin and improves insulin resistance in elderly men with and without type 2 diabetes mellitus: a randomised controlled clinical trial. Eur J Sport Sci. (2021) 21:636–45. doi: 10.1080/17461391.2020.1762755, PMID: 32345132

[36] Shakil-ur-Rehman S Karimi H Gillani SA. Effects of supervised structured aerobic exercise training program on fasting blood glucose level, plasma insulin level, glycemic control, and insulin resistance in type 2 diabetes mellitus. Pak J Med Sci. (2017) 33:576–80. doi: 10.12669/pjms.333.12023, PMID: 28811774 PMC5510106

[37] Farrokhian A Bahmani F Taghizadeh M Mirhashemi SM Aarabi MH Raygan F . Selenium supplementation affects insulin resistance and serum hs-CRP in patients with type 2 diabetes and coronary heart disease. Horm Metab Res. (2016) 48:263–8. doi: 10.1055/s-0035-1569276, PMID: 26743526

[38] Chiti H. The effects of 12-weeks combined exercises on cost, dosage of insulin, and glycemic indices in type 2 diabetic patients. J Adv Med Biomed Res. (2024) 32:191–201. doi: 10.61186/jambr.32.152.191

[39] Terauchi Y Takada T Yoshida S. A randomized controlled trial of a structured program combining aerobic and resistance exercise for adults with type 2 diabetes in Japan. Diabetol Int. (2022) 13:75–84. doi: 10.1007/s13340-021-00506-5, PMID: 35059244 PMC8733075

[40] Jeon YK Kim SS Kim JH Kim HJ Kim HJ Park JJ . Combined aerobic and resistance exercise training reduces circulating apolipoprotein J levels and improves insulin resistance in postmenopausal diabetic women. Diabetes Metab J. (2020) 44:103–12. doi: 10.4093/dmj.2018.0160, PMID: 32097999 PMC7043986

[41] Hodaei H Adibian M Nikpayam O Hedayati M Sohrab G. The effect of curcumin supplementation on anthropometric indices, insulin resistance and oxidative stress in patients with type 2 diabetes: a randomized, double-blind clinical trial. Diabetol Metab Syndr. (2019) 11:1–8. doi: 10.1186/s13098-019-0437-7, PMID: 31149032 PMC6537430

[42] Khalili L Alipour B Jafar-Abadi MA Asghari Jafar-Abadi M Faraji I Hassanalilou T . The effects of *Lactobacillus casei* on glycemic response, serum sirtuin1 and fetuin-a levels in patients with type 2 diabetes mellitus: a randomized controlled trial. Iran Biomed J. (2019) 23:68–77. doi: 10.29252/ibj.23.1.68 29803203 PMC6305821

[43] Crochemore ICC Souza AF de Souza ACF Rosado EL. ω −3 polyunsaturated fatty acid supplementation does not influence body composition, insulin resistance, and lipemia in women with type 2 diabetes and obesity. Nutr Clin Pract. (2012) 27:553–60. doi: 10.1177/0884533612444535 22661243

[44] Li FX. Research on the clinical efficacy of Baduanjin combined with resistance exercise in patients with type 2 diabetes Guangzhou University of Chinese Medicine (2020).

[45] Salarinia M Azizi M Tahmasebi W Khalvandi H. Effect of eight weeks of vitamin D supplementation and water-based exercise on cardiometabolic profile in women with type 2 diabetes. Sci Sports. (2023) 38:283–92. doi: 10.1016/j.scispo.2022.04.008

[46] Dadrass A Mohamadzadeh Salamat K Hamidi K Azizbeigi K. Anti-inflammatory effects of vitamin D and resistance training in men with type 2 diabetes mellitus and vitamin D deficiency: a randomized, double-blinded, placebo-controlled clinical trial. J Diabetes Metab Disord. (2019) 18:323–31. doi: 10.1007/s40200-019-00416-z, PMID: 31890657 PMC6914746

[47] Hoseini R Rahim HA Ahmed JK. Concurrent alteration in inflammatory biomarker gene expression and oxidative stress: how aerobic training and vitamin D improve T2DM. BMC Complement Med Ther. (2022) 22:165. doi: 10.1186/s12906-022-03645-7, PMID: 35733163 PMC9214191

[48] El Hajj C Walrand S Helou M Yammine K. Effect of vitamin D supplementation on inflammatory markers in non-obese Lebanese patients with type 2 diabetes: a randomized controlled trial. Nutrients. (2020) 12:2033. doi: 10.3390/nu12072033, PMID: 32659891 PMC7400886

[49] Zhou WP Li LF Zhang WH. Clinical study on the combined Chinese medicinal exercise and conventional nursing intervention in type 2 diabetes. New Chin Med. (2020) 52:188–90. doi: 10.13457/j.cnki.jncm.2020.08.057

[50] El-Khodary NM Dabees H Werida RH. Folic acid effect on homocysteine, sortilin levels and glycemic control in type 2 diabetes mellitus patients. Nutr Diabetes. (2022) 12:33. doi: 10.1038/s41387-022-00210-6, PMID: 35732620 PMC9217798

[51] Rezagholizadeh F Keshavarz SA Djalali M Rad EY Alizadeh S Javanbakht MH. Vitamin D3 supplementation improves serum SFRP5 and Wnt5a levels in patients with type 2 diabetes: a randomized, double-blind, placebo-controlled trial. Int J Vitam Nutr Res. (2019) 89:341–50. doi: 10.1024/0300-9831/a000598 30856079

[52] Safarpour P Daneshi-Maskooni M Vafa M Nourbakhsh M Janani L Maddah M . Vitamin D supplementation improves SIRT1, Irisin, and glucose indices in overweight or obese type 2 diabetic patients: a double-blind randomized placebo-controlled clinical trial. BMC Fam Pract. (2020) 21:1–10. doi: 10.1186/s12875-020-1096-3, PMID: 32033527 PMC7007689

[53] Lai MH. Antioxidant effects and insulin resistance improvement of chromium combined with vitamin C and E supplementation for type 2 diabetes mellitus. J Clin Biochem Nutr. (2008) 43:191–8. doi: 10.3164/jcbn.2008064, PMID: 19015754 PMC2581761

